# Hepatic Transcriptome Analysis Identifies Divergent Pathogen-Specific Targeting-Strategies to Modulate the Innate Immune System in Response to Intramammary Infection

**DOI:** 10.3389/fimmu.2020.00715

**Published:** 2020-04-29

**Authors:** Annika Heimes, Johanna Brodhagen, Rosemarie Weikard, Hans-Martin Seyfert, Doreen Becker, Marie M. Meyerholz, Wolfram Petzl, Holm Zerbe, Martina Hoedemaker, Laura Rohmeier, Hans-Joachim Schuberth, Marion Schmicke, Susanne Engelmann, Christa Kühn

**Affiliations:** ^1^Leibniz Institute for Farm Animal Biology (FBN), Institute of Genome Biology, Dummerstorf, Germany; ^2^Clinic for Ruminants with Ambulatory and Herd Health Services, Centre for Clinical Veterinary Medicine, Ludwig-Maximilians-University Munich, Oberschleißheim, Germany; ^3^Clinic for Cattle, University of Veterinary Medicine Hanover, Hanover, Germany; ^4^Clinic for Swine, Small Ruminants, Forensic Medicine and Ambulatory Service, University of Veterinary Medicine Hanover, Hanover, Germany; ^5^Immunology Unit, University of Veterinary Medicine Hanover, Hanover, Germany; ^6^Faculty of Natural Sciences III, Martin-Luther Universität Halle-Wittenberg, Halle, Germany; ^7^Technical University Braunschweig, Institute for Microbiology, Brunswick, Germany; ^8^Helmholtz Centre for Infection Research, Microbial Proteomics, Brunswick, Germany; ^9^Agricultural and Environmental Faculty, University Rostock, Rostock, Germany

**Keywords:** mastitis, bovine, *S. aureus*, *E. coli*, transcriptome, liver

## Abstract

Mastitis is one of the major risks for public health and animal welfare in the dairy industry. Two of the most important pathogens to cause mastitis in dairy cattle are *Staphylococcus aureus* (*S. aureus*) and *Escherichia coli* (*E. coli*). While *S. aureus* generally induces a chronic and subclinical mastitis, *E. coli* is an important etiological pathogen resulting in an acute and clinical mastitis. The liver plays a central role in both, the metabolic and inflammatory physiology of the dairy cow, which is particularly challenged in the early lactation due to high metabolic and immunological demands. In the current study, we challenged the mammary glands of Holstein cows with *S. aureus* or *E. coli*, respectively, mimicking an early lactation infection. We compared the animals' liver transcriptomes with those of untreated controls to investigate the hepatic response of the individuals. Both, *S. aureus* and *E. coli* elicited systemic effects on the host after intramammary challenge and seemed to use pathogen-specific targeting strategies to bypass the innate immune system. The most striking result of our study is that we demonstrate for the first time that *S. aureus* intramammary challenge causes an immune response beyond the original local site of the mastitis. We found that in the peripheral liver tissue defined biological pathways are switched on in a coordinated manner to balance the immune response in the entire organism. TGFB1 signaling plays a crucial role in this context. Important pathways involving actin and integrin, key components of the cytoskeleton, were downregulated in the liver of *S. aureus* infected cows. In the hepatic transcriptome of *E. coli* infected cows, important components of the complement system were significantly lower expressed compared to the control cows. Notably, while *S. aureus* inhibits the cell signaling by Rho GTPases in the liver, *E. coli* switches the complement system off. Also, metabolic hepatic pathways (e.g., lipid metabolism) are affected after mammary gland challenge, demonstrating that the liver restricts metabolic tasks in favor of the predominant immune response after infection. Our results provide new insights for the infection-induced modifications of the dairy cow's hepatic transcriptome following mastitis.

## Introduction

In times of growing consumer awareness, the delivery of high-quality products from healthy animals is a high priority for animal farming and the food industry. One major potential risk for public health and animal welfare in the dairy industry is mastitis, the infection and inflammation of the mammary gland. It is one of the most frequent and costly single-animal diseases in dairy cows (1), raises concerns in human and veterinary medicine due to potentially increasing resistance of pathogens against antimicrobial drugs (2, 3) and, if left untreated, can seriously damage the animal's health (4).

One approach to reduce the incidence of infections in animal production is the breeding of individuals with lower susceptibility to infectious diseases (5). For this purpose, it is important to deeply phenotype the individuals for the target traits when searching for the causal background of the genetic variation. Two of the most important pathogens to cause mastitis in dairy cattle are *Staphylococcus aureus* (*S. aureus*) and *Escherichia coli* (*E. coli*). While *S. aureus* generally induces a chronic and subclinical mastitis (6), *E. coli* is an important etiological pathogen resulting in an acute and clinical mastitis (7). Furthermore, *S. aureus* is Gram-positive, whereas *E. coli* is Gram-negative (7). The pathogenesis and infection dynamics of the respective forms of mastitis differ significantly depending on the pathogen type. Gram-negative bacteria release endotoxin or lipopolysaccharide (LPS), a component of their cell wall (6, 7). Many studies consider LPS and its fractions to be a potent factor of the *E. coli* induced mastitis pathogenesis (7, 8). But it has to be recognized that peripheral LPS and whole pathogen challenge can elicit differential responses (9). Gram-positive bacteria (e.g., *S. aureus)* rely on completely different virulence factors, e.g., exotoxins (6, 10). Whereas the mammary gland response to these pathogens is increasingly well understood, the consequences for peripheral tissues are less thoroughly investigated.

Thus, in our comprehensive network project, we pursued a holistic approach to mastitis in order to obtain a deeper understanding of the underlying biological networks affected in response to an intramammary challenge with mastitis pathogens at an early stage of lactation associated with particularly high disease incidence. A part of this study had a particular focus on the response of the hepatic transcriptome of cows whose mammary glands were experimentally challenged with live *S. aureus* or *E. coli* compared to non-challenged cows. The liver is an important organ as it plays a central role in both, the metabolic and inflammatory physiology of the dairy cow (11). Given the particularly heavy metabolic hepatic workload in early lactation of dairy cows (12), any alteration or impairment of liver function might have detrimental effects on animal health in this critical period. Our hypothesis is that the elucidation of the interaction between metabolism and immune response in the liver of cows might provide new insights into the disease-associated hepatic processes in early lactation. This will open new potential perspectives for the prevention and treatment of mastitis and contribute to the discovery of biomarkers for mastitis incidence.

In our study, we have placed a special focus on the response of the liver transcriptome to *S. aureus* infection, which is commonly assumed to have no or only rare systemic effects on the host after intramammary infection (13–15). In contrast, *E. coli* infections are well-known for their systemic effects (7, 13, 16). There are also studies that previously investigated the hepatic transcriptome after experimentally induced *E. coli* mammary gland infection (11, 17–19). However, to our best knowledge, there are no reports comparing the hepatic transcriptome of cows after an *S. aureus* mammary gland challenge with that of cows challenged with *E. coli* and that of non-challenged cows.

## Materials and Methods

### Animals and Study Design

For our study, we allocated 41 gestating Holstein cows in two experimental groups. Thirty-six of these animals were challenged intramammary with pathogens (either *S. aureus* or *E. coli*) in an infection model at the Clinic for Cattle at the University of Veterinary Medicine Hanover, in the following referred to as TiHo. The remaining five animals were brought to the Leibniz Institute for Farm Animal Biology in Dummerstorf, in the following referred to as FBN, to serve as a non-challenged control and to participate in a long-term model. The husbandry and sample collection of both experimental groups were previously described (20–22). For the TiHo cohort, the experiment was approved under the reference number 33.12-42502-04-15/2024 by the Lower Saxony Federal State Office for Consumer Protection and Food Safety. For the FBN cohort, the experiment was approved under the reference number 7221.3-1-055/15 by the responsible authority (LALLF, Landesamt für Landwirtschaft, Lebensmittelsicherheit und Fischerei Mecklenburg-Vorpommern, Rostock, Germany). Furthermore, this study was submitted to and approved by the ethics committees of the University of Veterinary Medicine Hanover and the Leibniz Institute for Farm Animal Biology, respectively. All ethical evaluations were performed as required by the German Animal Care Law and associated legislative regulations (23).

**Figure 1:**
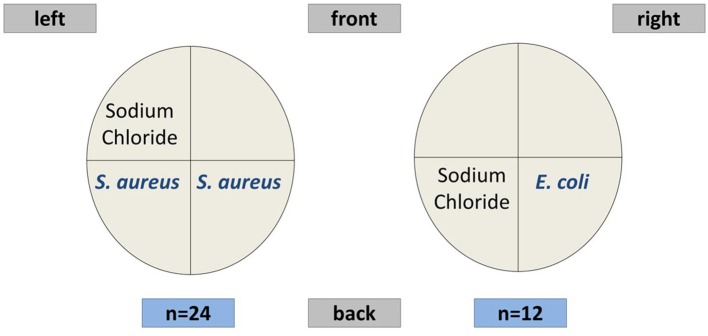
Scheme of the mammary gland quarters in the pathogen challenge experiment.

The challenge scheme for the TiHo cohort followed an established and well-studied model (24, 25). In week 6, at 36 ± 3 days after parturition, 24 healthy animals were challenged with 10,000 CFU (colony forming units) *S. aureus* 1027 each in both hind quarters of the mammary gland. Twelve animals were challenged with 500 CFU *E. coli* 1303 in one hind quarter of the mammary gland. Both challenge groups had a control udder quarter infused with sterile sodium chloride solution. The infection doses had been established in previous experiments of our group and had already been applied in a number of studies (13, 25). According to the experience from those studies, the doses were selected to successfully induce a clinical mastitis of *S. aureus* or *E. coli*, respectively. A schematic overview of the intramammary challenge scheme is shown in Figure 1. After challenge, body temperature of all animals was monitored by an intravaginal logger.

Prior to morning milking on day 10 a.p., 2 p.p., 7 p.p., 14 p.p., 21 p.p., and in week 6, blood samples were collected from the *Vena jugularis*. Serum concentrations of NEFA (non- esterified fatty acids) and BHB (*beta*-hydroxybutyric acid) were determined using the ABX Pentra 400 (HORIBA, Ltd., Kyoto, Japan). Differences between cohorts (TiHo vs. FBN) were evaluated in a Mixed linear model with fixed effects of day of sampling, cohort, and day of sampling × cohort interaction and a random animal effect with the lme4 package in R (26).

The cows challenged with *S. aureus* were sacrificed 96 h after challenge, whereas the cows challenged with *E. coli* were killed 24 h after challenge (captive bolt gun followed by immediate exsanguination). These time points were related to the different peaks of inflammation in the mammary gland after *S. aureus* or *E. coli* challenge, according to previous studies (24, 25). The collection of liver tissue samples of the TiHo cohort was carried out during the section of the animals. In the long-term model (FBN cohort), we collected liver samples immediately after captive bolt gun followed by immediate exsanguination at a lactation stage analogous to cows of the TiHo cohort, 6 weeks in their second lactation. The liver tissue samples were dissected from the liver *Lobus caudatus*, immediately shock frozen in liquid nitrogen, and subsequently stored at −80°C.

### Transcriptome Analysis by RNA Sequencing

The frozen liver tissue samples (approximately 30 mg) were grinded using the Precellys 24 tissue homogenizer (peQLab, Erlangen, Germany). Total RNA was extracted using the NucleoSpin RNA II kit (Macherey-Nagel, Düren, Germany) via an on-column-purification including a modified DNase I digestion step as described by Weikard et al. (27). Subsequently, total RNA was checked for presence of genomic DNA by PCR (28), and additional DNase I digestion was applied when necessary. RNA concentration and purity were measured on a NanoDrop 2000 spectrophotometer (Thermo Fisher Scientific, Waltham, MA) and a Qubit 2.0 fluorometer (Thermo Fisher Scientific, Waltham, MA). RNA integrity was monitored on the Bioanalyzer 2100 (Agilent Technologies, Böblingen, Germany). For RNA sequencing (RNAseq) library preparation we followed a strand-specific protocol (TruSeq Stranded mRNA LP, Illumina, San Diego, CA) including polyA-selection. Indices were applied for multiplexing during cluster generation and sequencing. The RNAseq libraries were monitored for quality on the Bioanalyzer 2100. Paired end library sequencing (2 × 90 base pairs) was performed on the Illumina HiSeq 2500 system (Illumina, San Diego, CA).

All RNAseq datasets are submitted to the ENA repository (https://www.ebi.ac.uk/ena) and are publicly accessible under project number PRJEB33849, accession numbers ERR3466640 - ERR3466680 at EMBL-EBI.

### Bioinformatic Analysis

Demultiplexing was performed with CASAVA v1.8 (Illumina, San Diego, CA). For data processing, we used SAMtools (29) as well as Linux and R-based (30) scripts. Quality of the reads was controlled with FastQC version 0.11.5 (31) and MultiQC version 1.4 (32). We applied Cutadapt version 1.12 (33) to remove adapters and Qualitytrim (34) to eliminate low-quality bases. Read alignment to the bovine reference genome UMD3.1 with Ensembl 87 reference annotation (35) was carried out with Hisat2 version 2.1.0 (36). A guided transcript assembly was performed with StringTie version 1.3.2.d (36). This strategy considered the reference genome annotation and additionally allowed the assembly of reads for transcripts not yet annotated. Using the StringTie (36) merge function, we created a project specific annotation for read counting with featureCounts version 1.5.2 (37). For differential expression analysis we used DESeq2 version 1.18.1 (38). For the analysis, we only included loci with at least four samples with at least 11 reads assigned. For graphical displays of the results, the R packages ggplot2 (39), pheatmap (40), ComplexHeatmap v2.2.0 (41), and EnhancedVolcano (42) were used. To establish clusters of co-expressed genes, we performed a weighted gene co-expression network analysis (43) implemented in the R package WGCNA (44) version 1.68, including all expressed loci from control samples as well as samples from *E. coli* or *S. aureus* challenged animals. An adjacency matrix of pairwise correlations between expression levels of all pairs of genes across all samples was generated, reporting the connection strength between gene pairs. For constructing the weighted gene network, we selected a soft thresholding power β as calculated by the picSoft Threshold function. Saturation of the respective scale-free topology index was reached at a value of 7. Hierarchical clustering established network modules. For the module generating function, blockwise Modules, we selected a minimum module size of 30, and a threshold for merging modules of 0.25 while keeping all other parameters at default. We then searched for modules, which displayed a correlation to the challenge effects. To this end, we first calculated the proportion of genes, which showed differential expression for each comparison pair, control versus *S. aureus* and *E. coli* challenged animals, respectively. We established a spearman rank correlation between the module eigengene value for each sample and its infection group score (with 0 = control, 1 = *S. aureus*, 2 = *E. coli*) assuming no, mild or severe clinical response to pathogen. In our analyses, we furthermore calculated the module membership for each gene and module, which is the correlation between the normalized gene expressions and eigengene value of a sample. We plotted the module membership against the correlation between normalized gene expression and infection identifier (representing a measure of gene trait significance) within modules. For pathway analyses, it is assumed that genes, which are highly interconnected within a network eigengene module, should be involved in closely linked or identical biological pathways. Thus, for those modules showing a high correlation between the module eigengene value for each sample and its infection group score, differentially expressed genes (DEGs) by pathogen comparison were modulewise submitted to Ingenuity Pathway Analysis (IPA, Qiagen, Hilden, Germany) with a threshold for significance of *p*_adj_ < 0.05. In addition, we also tested for enriched KEGG pathways via DAVID (45). In the pathogen-specific analyses, we focused on those modules by pathogen challenge, which were in the highest quartile across all modules regarding the proportion of DEGs.

Finally, IPA pathway analyses were also performed for all DEGs in the comparison between samples from controls and *E. coli* or *S. aureus* challenged animals, respectively.

## Results

### Statistics and Distribution of Samples

RNAseq generated a total of 4.5 billion reads corresponding to an average of 110 million reads per liver sample. Ninety-eight percent of reads mapped at least once to the bovine reference genome UMD3.1 (35).

**Figure 2:**
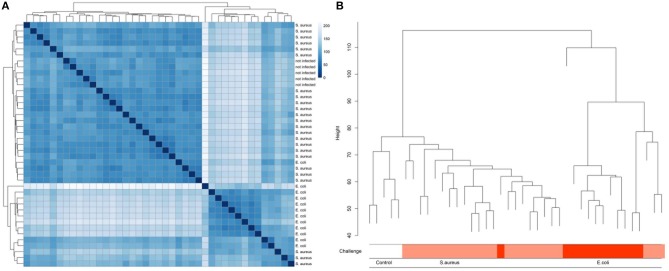
Heat map **(A)** and clustering **(B)** of liver transcriptome expression data from cows after experimental intramammary challenge with *S. aureus* and *E. coli* and non-challenged control cows.

A multidimensional scaling (MDS) plot represents the distribution of hepatic transcriptome data from cows having received an intramammary challenge with *S. aureus* and *E. coli* and non-challenged control samples along PC (principal component) 1 and PC2 (Supplementary Image 1). The results clearly demonstrate that there is an essential clustering of animals with respect to the pathogen challenge, but also a substantial variation of animals within the pathogen groups, particularly for the *E. coli* challenge. Single *E. coli* challenged animals cluster with *S. aureus* challenged individuals, while four animals challenged with *S. aureus* form an outgroup for PC1. The control group cows cluster together, but they are not distinct from the majority of *S. aureus* challenged cows. Similar results were obtained, when cluster analyses between samples were conducted (Figure 2). The cluster tree is essentially divided into two branches equivalent to *E. coli* or *S. aureus*/control animals. However, the *E. coli* branch of the cluster tree contains three animals challenged with *S. aureus*. The five control animals formed a subgroup within the branch of essentially *S. aureus* challenged animals. This is in line with data on serum NEFA and BHB. In the sixth lactation week (immediately before the challenge) there was no significant difference between individuals in the TiHo and FBN cohort [NEFA: TiHo: 388 μmol/l ± 44.1 (s.e.) FBN: 342 μmol/l ± 107.9 (s.e.), *p* = 0.69; BHB: TiHo: 0.457 mmol/l ± 0.078 (s.e.), FBN: 0.592 mmol/l ± 0.190 (s.e.), *p* = 0.51].

### Differential Expression Analysis

#### *S. aureus* Challenge

**Figure 3:**
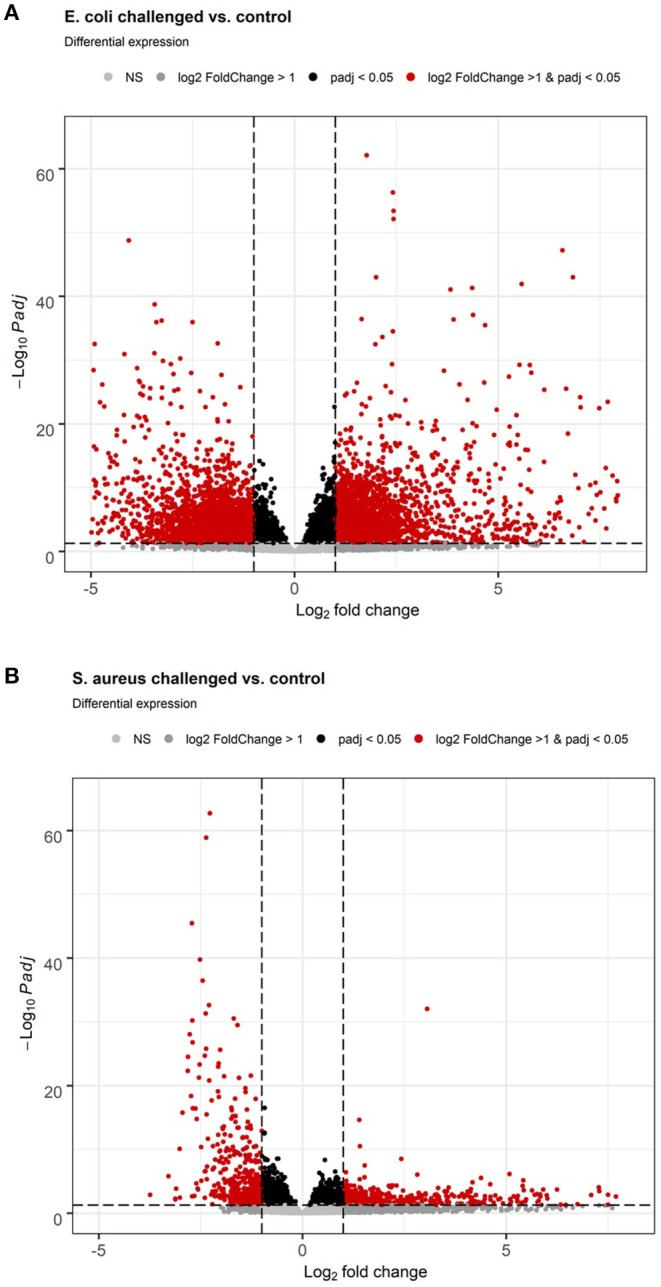
Volcano plot of differential gene expression analysis of the hepatic transcriptome between individuals with intramammary pathogen challenge and controls. **(A)** *E. coli* vs. control. **(B)** *S. aureus* vs. control.

*S. aureus* mammary gland infection is usually considered to be a subclinical inflammation with local pathological manifestation. However, the body temperature course data after the pathogen challenge monitored by the intravaginal logger indicated a clinical response to the challenge as demonstrated by a maximum mean body temperature exceeding 40.0°C in 83% of the *S. aureus* challenged animals during the course of challenge. This clinical pathogen response is supported by the hepatic transcriptome analysis, which revealed a total of 3,672 genes differently expressed between liver samples of *S. aureus* challenged cows compared to the control group (*p*_adj_ < 0.05, 2,021 lower and 1,651 higher expressed in *S. aureus*, see also Supplementary Table 1). Of these loci, 2,137 were annotated genes (58%). Several of the loci with very low *p*_adj_ values and high log2 Fold Changes are yet unannotated in the bovine genome and merit further investigation. More than 55% of the DEGs displayed a lower expression after *S. aureus* challenge compared to untreated controls. Although the number of DEGs in the liver in response to intramammary *S. aureus* challenge is substantial, it is lower than observed for the *E. coli* challenge. Comparison of the volcano plots from *S. aureus* vs. *E. coli* challenge (Figure 3) demonstrated the substantially stronger effect of *E. coli* compared to *S. aureus* challenge, particularly regarding strongly upregulated genes.

#### *E. coli* Challenge

*E. coli* mammary infection usually elicits a systemic inflammatory response. Correspondingly, all challenged cows developed fever (inner body temperature > 39.5°C) in the time period 24 h after challenge. The average maximum temperature of the animals was 41.8°C (22). This strong systemic response was also reflected in the liver transcriptome. When comparing the liver transcriptome of cows challenged with *E. coli* with that of the non-challenged control group, 10,648 loci were significantly differentially expressed (*p*_adj_ < 0.05, see Supplementary Table 2, Figure 3). Thus, more than 50% of all loci passing the threshold for being included in the differential expression analysis (20,723) were differentially expressed between the *E. coli* challenged cows and the control group. Of these significantly DEGs, 6,720 were annotated genes (63%). There was a tendency for more DEGs with higher expression in *E. coli* challenged animals than with lower expression compared to untreated controls (3,548 higher, 3,172 lower expressed; Figure 3).

### Module Analysis Searching for Clusters of Genes Associated With Pathogen Challenge

#### Data Analysis Across Pathogen Challenge Groups

To identify further mechanisms in the hepatic pathogen-specific response to intramammary infection, we performed a module-based cluster analysis across all samples. The respective WGCNA analysis yielded 31 distinct modules (Supplementary Table 3). We found 15 modules, which were significantly correlated with the infection category (0 = control, 1 = *S. aureus*, 2 = *E. coli*, Table 1). Six of those modules showed a negative and nine modules a positive correlation: Within the modules with significant correlation to the infection identifier *E. coli* eight modules were in the upper quartile across all modules regarding the proportion of DEGs (Table 1). For the infection identifier *S. aureus*, four significantly correlated modules were in the upper quartile across all modules regarding the proportion of DEGs. However, in contrast to the *E. coli* infection identifier, where all significantly correlated modules in the upper quartile regarding the proportion of DEGs were also significantly correlated to the infection identifier, half of the modules in the upper quartile did not show this pattern for the *S. aureus* category. Instead, for the *S. aureus* identifier, four modules without significant correlation to the infection category score were in the upper quartile regarding the proportion of DEGs. This applied particularly to the modules “yellow” and “grey60”. It has to be considered that three of those modules only harbored a limited number of annotated genes.

**Table 1:** Module characteristics after WGCNA analysis of the hepatic transcriptomes including individuals challenged intramammary with *E. coli* or *S. aureus* and unchallenged controls.

**Module name**	**Spearman correlation between module eigengene and. infection category^a^**	***p*_value^a^**	**N genes in module^b^**	**N DEGs *E. coli*^c^**	**Proportion of DEGs *E. coli*^d^**	**N DEGs *S. aureus*^e^**	**Proportion of DEGs *S. aureus*^f^**
Turquoise	−0.76	7.31E-09	6,067	4,067	0.67	1148	0.19
Darkred	−0.51	5.94E-04	71	50	0.70	9	0.13
Pink	−0.49	1.05E-03	406	216	0.53	37	0.09
Red	−0.41	7.83E-03	553	102	0.18	37	0.07
Darkorange	−0.40	9.83E-03	59	11	0.19	4	0.07
Royalblue	−0.33	3.27E-02	72	61	0.85	52	0.72
Steelblue	0.32	3.95E-02	37	11	0.30	0	0.00
Brown	0.35	2.29E-02	2,436	653	0.27	503	0.21
Gray	0.43	5.40E-03	685	57	0.08	44	0.06
Cyan	0.44	4.01E-03	182	30	0.16	23	0.13
Darkturquoise	0.51	7.00E-04	65	42	0.65	29	0.45
Midnightblue	0.51	6.77E-04	171	89	0.52	63	0.37
Tan	0.54	2.49E-04	197	84	0.43	36	0.18
Green	0.61	2.21E-05	1,001	515	0.51	178	0.18
Blue	0.67	1.31E-06	4,875	3,586	0.74	485	0.10
Magenta	−0.30	5.29E-02	258	49	0.19	26	0.10
Salmon	−0.29	6.81E-02	188	32	0.17	2	0.01
Darkgrey	0.28	7.33E-02	61	18	0.30	15	0.25
Purple	0.27	8.42E-02	235	31	0.13	25	0.11
Saddlebrown	0.25	1.08E-01	53	16	0.30	6	0.11
Yellow	−0.24	1.36E-01	1,751	753	0.43	678	0.39
Darkgreen	0.22	1.58E-01	69	1	0.01	12	0.17
Skyblue	−0.17	2.77E-01	55	5	0.09	0	0.00
Lightyellow	0.15	3.57E-01	80	17	0.21	12	0.15
Black	0.14	3.98E-01	436	68	0.16	76	0.17
White	−0.13	4.18E-01	58	4	0.07	12	0.21
Lightcyan	0.09	5.60E-01	145	10	0.07	15	0.10
Grey60	0.08	6.34E-01	96	42	0.44	90	0.94
Orange	0.07	6.54E-01	59	0	0.00	3	0.05
Lightgreen	0.03	8.74E-01	91	19	0.21	15	0.16
Greenyellow	0.00	9.90E-01	211	9	0.04	40	0.19

a p-value for spearman correlation between module eigengene and infection category by sample;

b Number of genes included in the respective module;

c Number of DEGs (p_adj_ < 0.05) between E. coli challenged and control animals;

d Proportion of genes in the respective module that are differentially expressed between E. coli challenged and control animals (modules with a proportion in the highest quartile indicated in red);

e Number of DEGs (padj <0.05) between S. aureus challenged and control animals;

f *Proportion of genes in the respective module that are differentially expressed between S. aureus challenged and control animals (modules with a proportion in the highest quartile indicated in red)*.

**Figure 4:**
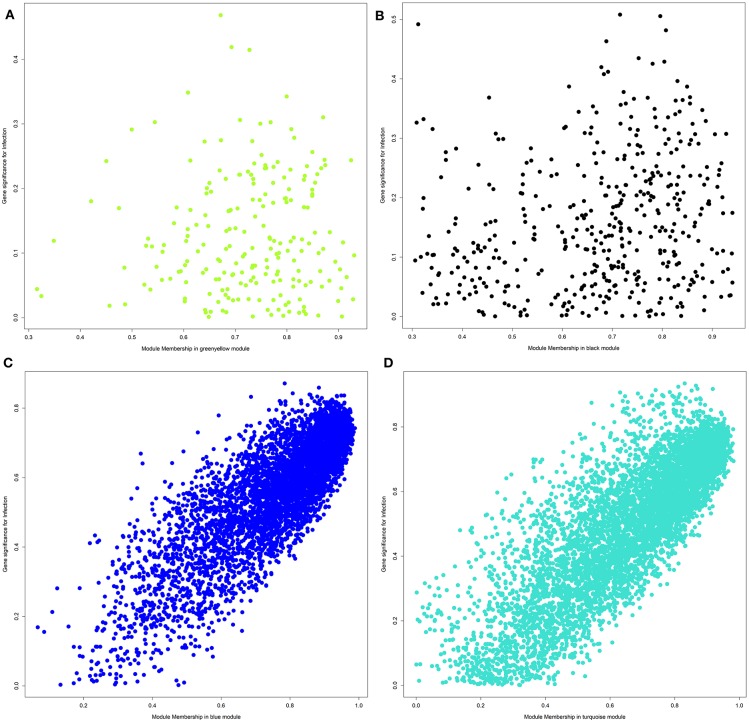
Plot of module membership against the correlation between normalized gene expression and infection category for each gene within a module. **(A)** Module greenyellow; **(B)** module black; **(C)** module blue; **(D)** module turquoise.

For each module and each gene, we then plotted the module membership measure (a parameter for how well a gene fits into the assigned module) against the correlation between normalized gene expression and infection category (representing a measure of gene-trait relationship significance). While modules with a significant correlation to the infection category showed a very clear linear relationship of those two parameters (see Figures 4C,D), we did not observe this pattern for uncorrelated modules (Figures 4A,B).

#### Data Analysis Within Pathogen Challenge Groups

For subsequent pathway analyses, we focused on those modules within the upper quartile across all modules regarding the proportion of DEGs and also on those with a significant correlation to the infection identifier. There were a number of modules with a significant correlation to the infection identifier, which demonstrated pathogen-challenge specific differences in the proportion of DEGs (Table 1). These modules were further analyzed within pathogen challenge groups by clustering analyses via heatmaps (Supplementary Images 2, 3) and were investigated for pathways and biological function GO terms enriched by DEGs.

#### *E. coli* Challenge

For *E. coli* challenge, pathway enrichment analysis highlighted the modules turquoise, darkred, pink, green and blue (Supplementary Image 2, Tables 1, 2, Supplementary Tables 4–6). These modules showed a clustering of samples for infection status and had a number of biological pathways and functions enriched with DEGs. The module turquoise by far harbored the largest number of genes in our dataset (Table 1) and showed pathways enriched with DEGs involved in Fatty Acid Beta Oxidation and Acute Phase response (Table 2, Supplementary Table 4). A particularly strong inactivation or activation according to the calculated *z*-scores was predicted for these two pathways. Accordingly, all members of the IPA Fatty Acid Beta Oxidation pathway were downregulated. DAVID enrichment analysis further revealed many KEGG pathways and biological function GO terms for fat and amino acid metabolism enriched with DEGs from this module (e.g., Valine, leucine, and isoleucine degradation, Fatty acid metabolism or PPAR signaling pathway, Supplementary Table 6). This fits in with the observation that several transcription factors known to be activators of fatty acid metabolism (e.g., PPARA, PPARG, PPARD) were found in the IPA analyses as upstream regulators with a predicted inhibition action status (Supplementary Table 5). Taken together, the data indicate that genes in this module are substantially involved in the inactivation of fatty acid metabolism and amino acid degradation highlighting the impaired metabolic function of the liver after intramammary *E. coli* challenge. The module is of particular interest, because it links the lipid metabolism with the immune response. The pathway Acute Phase response, one of the major tools of innate immune response is significantly enriched with DEGs from this pathway. Its positive *z*-score indicates its activation. The nuclear receptor HNF4A that, for example, regulates the metabolism in the liver, was the most prominent transcription factor among the upstream regulators and was predicted to be strongly inactivated (Supplementary Table 5). This is consistent with its known role of being involved in both the metabolic and the immune-related functions of the hepatocytes. In contrast, the effects described for DEGs in the turquoise module were not observed for *S. aureus* challenge, and this module was not in the highest quartile regarding the proportion of DEGs for *S. aureus*.

**Table 2:** Summary of ingenuity and DAVID enrichment analysis with DEGs from WGCNA modules within pathogen challenge groups.

**Module name**	***p*_value**	**Module enriched with DEGs from pathogen challenge**		**IPA pathways**	**Top 3 IPA predicted upstream regulators**	**KEGG and GO biological pathways**
Turquoise	7.31E-09	*E. coli*		Fatty acid beta oxidation (inactivated), acute phase response (activated)	HNF4A (–), XBP1 (+), PPARA (–)	Valine, leucine, and isoleucine degradation, fatty acid metabolism, PPAR signaling pathway
Darkred	5.94E-04	*E. coli*		Cholesterol biosynthesis (inactivated)	SREBF2 (–), SCAP (–), SREBF1 (–)	Lipid synthesis, steroid biosynthesis
Pink	1.05E-03	*E. coli*		PKCθ, signaling in T lymphocytes, Th1 pathway (inactivated), th2 pathway (inactivated)	IL2, CD3, LPS	Immune response, primary immunodeficiency, T cell receptor signaling
Royalblue	3.27E-02	*E. coli*	*S. aureus*	*E. coli* and *S. aureus:* Wnt/β-catenin signaling	*E. coli* and *S. aureus:* SMURF2, TNKS2, YY1AP1	*E. coli* and *S. aureus*: n.s.
Brown	2.29E-02		*S. aureus*	Epithelial adherens junction signaling, RhoGDI signaling (activated), actin cytoskeleton signaling (inactivated)	MAPT, TP53, CLPP	FcgammaR mediated phagocytosis
Darkturquoise	7.00E-04	*E. coli*	*S. aureus*	Apelin adipocyte signaling pathway	RBFOX2, ELAVL1, STOX1	*E. coli* and *S. aureus:* n.s.
Midnightblue	6.77E-04	*E. coli*	*S. aureus*	*E. coli* and *S. aureus*: urate biosynthesis/inosine 5′ -phosphate degradation	*E. coli*: MMP3, TFAP2E, JADRR; *S. aureus:* TFAP2E, JADRR, KCND2	*E. coli* and *S. aureus*: RNA processing, mRNA processing
Green	2.21E-05	*E. coli*		ERK5 signaling (activated), apoptosis Signaling (activated), phospholipase C signaling (activated)	HNF4A, NUPR1, POU5F1	n.s.
Blue	1.31E-06	*E. coli*		RAN signaling (activated), complement pathway (inactivated)	HNF4A, CST5, PHF12	RNA processing, RNA degradation, complement and coagulation cascade
Yellow	1.36E-01		*S. aureus*	ILK signaling (inactivated), Fcγ receptor-mediated phagocytosis in macrophages and monocytes (inactivated), IL-8 signaling (inactivated)	TGFB1, IFNG, TNF	Cytoskeleton organization, focal adhesion

*For further details see Supplementary Tables 4–9*.

The darkred module contained only 71 genes (Table 1, Supplementary Table 4) but also displayed a particular cluster of genes involved in metabolism. The dominant feature in the enrichment analysis of this module was the Cholesterol Biosynthesis pathway, which was observed as an inactivated pathway in IPA and was supported by corresponding biological function GO terms and KEGG pathway data from DAVID analysis (Table 2, Supplementary Tables 4, 6). Predicted upstream regulators for DEGs in this module were, e.g., *SREBF1* and *SREBF2*, which were assigned an inactivated status (Supplementary Table 5). They are known to be important for cholesterol homeostasis by regulating the transcription of sterol-regulated genes, and in our study, they were highly significantly (*p*_adj_. = 0.0003 and 0.0002 for *SREBF1* and *SREBF2*, respectively) lower expressed in *E. coli* challenged animals compared to controls. However, they themselves were not assigned to this module, but to the turquoise module.

In contrast to the turquoise and darkred modules, the pink module comprises DEGs, which were predominantly enriched in pathways and biological function GO terms associated with immune response, namely PKCθ Signaling in T Lymphocytes and Th1 and Th2 pathways (Table 2, Supplementary Tables 4, 6). Interestingly, almost all pathways for this module showed an inactivation status, this was most evident for the Th1 pathway. Suppressed hepatic response to *E. coli* challenge in the mammary gland is consistent with the prediction that upstream regulators with anti-inflammatory and attenuating cytokine signaling role like IL10 or SOCS1 are activated in the liver, and immune response stimulating factors like IL2 and IFNG are inactivated (Supplementary Table 5). Thus, this module provides a first indication on the inhibition of at least some sections of the hepatic immune response to intramammary *E. coli* challenge.

The module blue was another large module with >4,800 associated genes (Table 1), of which 2,386 were DEGs in the *E. coli* challenge (Table 2). In this module, we found the pathways and biological function GO terms related to protein and RNA processing significantly enriched with DEGs (Supplementary Tables 4, 6). Interestingly, also the Complement pathway, a central component of the innate immune response, was highlighted, because it had the lowest *z*-score of all enriched pathways indicating that this pathway is inactivated in *E. coli* challenged animals.

With 1,001 assigned genes in total, the module green (Table 1) was also a major module in size and provided also a conclusive clustering of *E. coli* challenged and control animals (Supplementary Image 2). However, despite >500 DEGs, only 14 IPA pathways and no KEGG pathway or biological function GO terms showed any significant enrichment with DEGs (Table 2, Supplementary Table 4). Thus, the specific function of the DEGs in this module is inconclusive, although, e.g., the ERK signaling pathway displayed enrichment.

#### *S. aureus* Challenge

For *S. aureus* challenge, the modules brown, yellow, royalblue, and grey60 were of particular interest, because they had a proportion of DEGs within the highest quartile across all modules and/or showed a clustering of samples for infection status (Supplementary Image 3, Tables 1, 2). In addition, except royalblue, they were not highly enriched with DEGs in the *E. coli* challenge compared to all other modules (Table 2).

The module brown is the largest module, which comprised a high proportion of DEGs for *S. aureus* challenge compared to all modules (Tables 1, 2). It showed an enrichment of DEGs for the IPA Epithelial Adherens Junction, Rho GDI signaling and Actin Cytoskeleton Signaling pathways (Supplementary Table 7). While for the Rho GDI signaling pathway activation was predicted, Actin Cytoskeleton signaling showed a highly negative *z*-score indicating inactivation. The DAVID analysis added further confirmation that DEGs in this module are involved in immune-related regulation of the actin cytoskeleton (Supplementary Table 9). The KEGG pathway FcγR mediated phagocytosis was significantly enriched with DEGs from this module. In the process of bacterial phagocytosis, FcγR crosslinking is known to induce changes in the actin cytoskeleton. Pathway enrichment analogous to the brown module was not observed in the *E. coli* challenge associated modules (Table 2).

For the yellow module, enrichment analyses with DEGs from the *S. aureus* challenge highlighted the ILK signaling, Fcγ Receptor mediated Phagocytosis in Macrophages and Monocytes, and IL8 signaling within the total of 118 enriched IPA pathways (Supplementary Table 7). All three had a negative *z*-score indicating inactivation of these pathways after *S. aureus* challenge. The major upstream regulators predicted for the DEGs in this module (TGFB1, IFNG, TNF, Supplementary Table 8) provide a direct link between the indicated pathways and an immune response to the pathogen.

Further modules, which display a clustering of samples by infection status are royalblue and grey60 (Supplementary Image 3). Yet, pathway analysis did not provide meaningful data for these modules due to a low number of annotated DEGs (Tables 1, 2). Nevertheless, particularly the module grey60 merits future further analysis due to a substantial number of genes with highly significant differential expression and high log2 FoldChange. Further attempts for improved functional annotation will be conducted, e.g., within the global initiative Functional Annotation of Animal Genomes (FAANG, www.faang.org).

### Pathway Enrichment Analysis of All DEGs After Intramammary *S. aureus* Challenge

When interpreting the results, it has to be considered that we have monitored the response of the animals in one tissue while the direct pathogen contact took place in another tissue. Thus, regulatory signals might not be directly measured at gene expression level in the liver, e.g., if the regulatory signal is a cytokine produced by blood monocytes. This is different to *in vitro* challenges, where only those signaling pathways that are active within the respective cell are monitored by expression analysis. Furthermore, we decided to analyze the DEGs across all modules within a pathogen challenge, because the analysis of genes by cluster has the limitation that a gene can only be member of a single cluster, but is involved in multiple pathways. This is demonstrated e.g., by SREB1 and SREB2, which were predicted as upstream regulators of DEGs after *E. coli* challenge in the module darkred. However, they themselves were assigned to another module (turquoise). Thus, we complemented the cluster-based data analysis with pathway analyses across all DEGs with pathogen challenge, *E. coli* or *S. aureus*.

The Ingenuity Pathway Analysis (IPA) revealed a substantial number of 124 significantly enriched canonical pathways in the liver transcriptome in response to intramammary *S. aureus* challenge (see Supplementary Table 10). Analogous to the modulewise pathway analyses, the main hepatic processes affected by *S. aureus* challenge are (i) Epithelial Adherens Junctions and Actin Cytoskeleton Signaling, (ii) ILK Signaling and Integrin Signaling, and (iii) RhoGDI Signaling (Table 3).

**Table 3:** Top 25 significantly enriched Ingenuity canonical pathways in the hepatic transcriptome comparing *S. aureus* challenged to non-challenged cows (NaN, no activation score).

**Ingenuity canonical pathways**	**–log (*p*-value)**	**Ratio**	***z*-score**
Epithelial adherens junction signaling	6.62E00	2.23E-01	NaN
ILK signaling	6.13E00	1.96E-01	−3.33
Axonal guidance signaling	5.87E00	1.53E-01	NaN
Germ cell-sertoli cell junction signaling	5.73E00	1.99E-01	NaN
Integrin signaling	5.42E00	1.82E-01	−4.23
Acute myeloid leukemia signaling	4.83E00	2.29E-01	−2.98
Sertoli cell-sertoli cell junction signaling	4.76E00	1.83E-01	NaN
RhoGDI signaling	4.51E00	1.8E-01	3.02
Hepatic fibrosis/hepatic stellate cell activation	4.51E00	1.78E-01	NaN
IL-8 signaling	4.41E00	1.74E-01	−4.70
Breast cancer regulation by Stathmin1	4.33E00	1.71E-01	NaN
ERK/MAPK signaling	4.28E00	1.72E-01	−1.71
Actin cytoskeleton signaling	4.18E00	1.65E-01	−4.56
PTEN signaling	4.11E00	1.98E-01	3.27
Fcγ receptor-mediated phagocytosis in macrophages and monocytes	4.11E00	2.12E-01	−4.15
Signaling by Rho family GTPases	3.93E00	1.57E-01	−4.80
Remodeling of epithelial adherens junctions	3.74E00	2.32E-01	NaN
Chronic myeloid leukemia signaling	3.67E00	1.98E-01	NaN
Reelin signaling in neurons	3.62E00	2.07E-01	NaN
Ephrin receptor signaling	3.62E00	1.69E-01	−3.92
VEGF signaling	3.49E00	1.93E-01	−2.98
FAK signaling	3.46E00	1.96E-01	NaN
CDK5 signaling	3.40E00	1.94E-01	−0.45
Tight junction signaling	3.38E00	1.68E-01	NaN
Adipogenesis pathway	3.36E00	1.78E-01	NaN

It was striking that numerous significantly enriched pathways were involved in the organization and maintenance of the cytoskeleton and/or intracellular or cell to cell signaling (e.g., Epithelial Adherens Junction Signaling, ILK Signaling, Integrin Signaling, RhoGDI Signaling, ERK/MAPK Signaling, Actin Cytoskeleton Signaling, Signaling by Rho family GTPases, Remodeling of Epithelial Adherens Junctions, FAK Signaling, and Tight Junction Signaling). All pathways with an indicated activation status in the Top 25 list of significantly enriched Ingenuity canonical pathways were inactivated except for RhoGDI Signaling and PTEN Signaling (Table 3).

#### Epithelial Adherens Junctions and Actin Cytoskeleton Signaling

**Figure 5:**
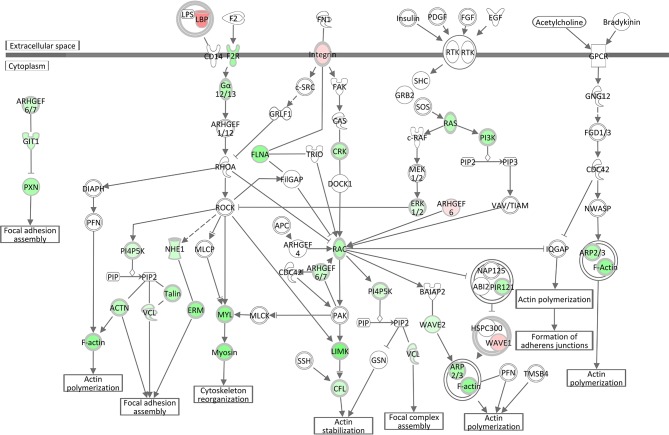
Gene expression in the actin cytoskeleton signaling pathway for the hepatic transcriptome of *S. aureus* challenged animals compared to the control liver transcriptome: green = lower expression and red = higher expression in *S. aureus* challenged animals compared to untreated control (adapted from IPA, Qiagen).

Analogous to the *S. aureus* associated module brown, Epithelial Adherens Junction Signaling was the most significantly affected pathway (see Table 3, Supplementary Image 4) in the liver of *S. aureus* challenged cows, which is associated with the also modified pathways Remodeling of Epithelial Adherens Junctions and Tight Junction Signaling. All but one of the 33 significantly DEGs allocated in this pathway were lower expressed in response to intramammary *S. aureus* challenge compared to control animals. Epithelial adherens junctions, also known as the cadherin/catenin complex, are the most important regulators of the actin cytoskeleton (46, 47), which alongside the tight junctions, function as principle mediators of cell-cell adhesion. The Actin Cytoskeleton Signaling itself had a negative *z*-score (−4.56) in our *S. aureus* challenge study, which reflects its substantial inactivation in infected animals (see Table 3, Figure 5). This pathway plays an important role in dynamic processes such as cell motility, axon guidance, cytokinesis, and phagocytosis. Its inactivation is consistent with the analogous negative *z*-scores also for ILK Signaling and Integrin Signaling (see below), which we had also observed for the *S. aureus* associated module yellow.

#### ILK Signaling and Integrin Signaling

**Figure 6:**
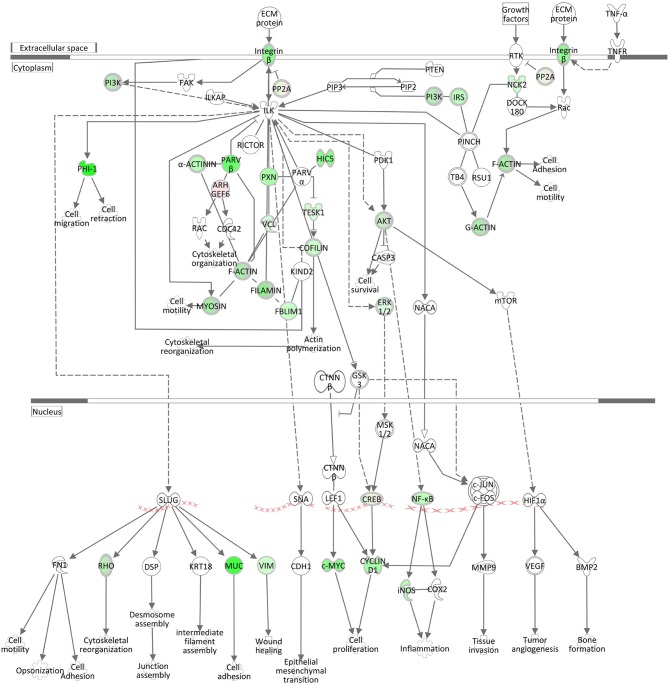
Gene expression of the ILK Signaling pathway in the hepatic transcriptome of *S. aureus* challenged animals compared to the control liver transcriptome: green = lower expression and red = higher expression in *S. aureus* challenged animals compared to untreated control (adapted from IPA, Qiagen).

The negative *z*-scores (−3.33) for the ILK (integrin-linked kinase) Signaling pathway and for the Integrin Signaling pathway (−4.23) show that both pathways were clearly inactivated in the liver of cows intramammary challenged with *S. aureus* (Table 3, Figure 6, Supplementary Image 5). The ILK protein is responsible for the conjunctions between integrins and the actin cytoskeleton (48), providing the link between the two pathway complexes. As indicated in Figure 6, ILK signaling mediated by *beta* integrin is central to a cascade of downstream processes including cell adhesion and cytoskeletal reorganization. Correspondingly to ILK Signaling, the Integrin Signaling pathway (Supplementary Image 5) displays significant enrichment of DEGs. While *alpha* integrins show a higher expression in the liver of *S. aureus* challenged cows compared to controls, *beta* integrin expression displays an opposite direction and is obviously a down-regulating driver for the inactivated ILK and Integrin signaling cascades.

#### Signaling by Rho Family GTPases and RhoGDI Signaling

**Figure 7:**
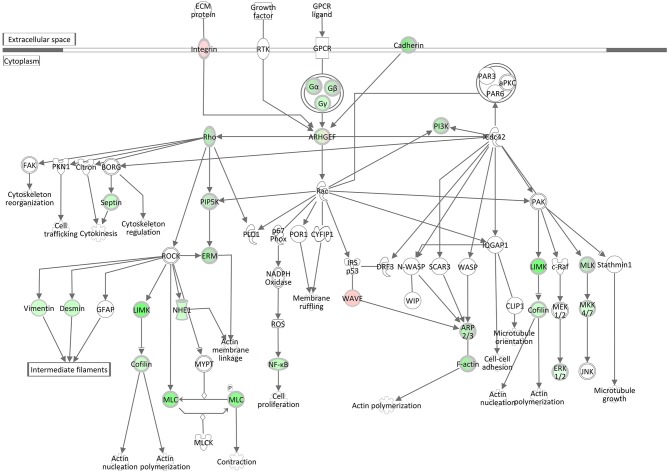
Gene expression of the signaling by rho family GTPases pathway in the hepatic transcriptome of *S. aureus* challenged animals compared to the control liver transcriptome: green = lower expression in *S. aureus* challenged animals compared to untreated control; red = higher expression in *S. aureus* challenged animals compared to untreated control (adapted from IPA, Qiagen).

Rho family GTPases are known to function as molecular switches that regulate a variety of biological processes, including cell proliferation, apoptosis, differentiation, migration, cytoskeletal reorganization, and membrane trafficking. The pathway Signaling by Rho Family GTPases was assigned a *z*-score of −4.8, the lowest activation score when comparing the hepatic transcriptome of *S. aureus* challenged cows to the control group (Table 3, Supplementary Table 10). This pathway was also enriched for DEGs in the *S. aureus* associated module brown. The low *z*-score indicates that genes involved in this pathway were considerably downregulated in response to intramammary challenge. Of the total 254 molecules included in this IPA pathway, 40 could be found as significantly differentially expressed in our analysis. Except for three genes, all displayed a downregulated gene expression level in response to *S. aureus* challenge (see also Supplementary Table 11). As presented in Figure 7, this pathway is associated with a variety of cytoskeletal processes affecting rearrangements of the plasma-membrane-associated actin cytoskeleton, cellular proliferation, and cytokinesis in liver tissue in response to the intramammary *S. aureus* challenge. This is consistent with the findings regarding the downregulation of the pathways Epithelial Adherens Junction Signaling, ILK Signaling, Integrin Signaling, and Actin Cytoskeleton Signaling (see above) but also with the upregulation of the RhoGDI Signaling pathway, which was assigned a positive *z*-score of 3.02. The RhoGDI (Rho GDP-dissociation inhibitor) is described as an inhibitor of Rho family GTPases activity by hampering the dissociation of GDP, preventing the subsequent binding of GTP (49). Thus, the activation of this network is plausible in the context of our analysis.

In summary, the results from the pathway analysis within the *S. aureus* associated modules as well as across all genes point toward a key role of actin cytoskeleton processes in the liver after intramammary *S. aureus* challenge.

### Analysis of Potential Drivers and Upstream Regulators in the Liver in Response to Intramammary *S. aureus* Challenge

Pathway analysis had highlighted β integrin at the intersection of the enriched pathways after *S. aureus* challenge (see above). To obtain information on the further transmission of the initial signals, the potential upstream regulators were monitored, which had been predicted for the DEGs by IPA. In response to *S. aureus* challenge, miRNAs were the most prominent upstream regulators in the hepatic transcriptome with the highest positive activation *z*-scores (Supplementary Table 12). Within the top 15 predicted upstream regulators with the lowest (negative) activation *z*-score is TGFB1. Because TGFB1 is known for its function in regulating immune responses and involvement in mastitis (13), it is a striking regulatory candidate. It was also by far the most significant upstream regulator predicted for DEGs in the *S. aureus*-associated module yellow. At transcription level, *TGFB1* showed a highly significant lower expression in *S. aureus* infected cows compared to control cows (Supplementary Table 1), which supports its predicted regulatory function.

### Pathway Enrichment Analysis of All DEGs After Intramammary *E. coli* Challenge

Ingenuity pathway analysis across all DEGs in the *E. coli* challenge revealed a slightly higher number (148) of significantly enriched canonical pathways in the liver transcriptome (Supplementary Table 10) compared to *S. aureus* challenge (see above). However, the main pathways identified as enriched after *E. coli* intramammary challenge differed from those obtained from the analysis of *S. aureus* challenged animals. This is in line with the differences detected in the modulewise data analysis. Key hepatic processes affected by *E. coli* challenge and attributed the highest activation scores, are (i) RXR Activation (and associated pathways) together with lipid metabolism (Adipogenesis, Fatty Acid *Beta* Oxidation), and (ii) Acute Phase Response and the Complement System (Table 4, Supplementary Table 10).

**Table 4:** Top 25 significantly enriched Ingenuity canonical pathways in the hepatic transcriptome comparing *E. coli* challenged to non-challenged cows (NaN, no activation score).

**Ingenuity canonical pathways**	**–log (*p*-value)**	**Ratio**	***z*-score**
Protein ubiquitination pathway	9.44E+00	4.42E-01	NaN
Sirtuin signaling pathway	8.41E+00	4.15E-01	−1.43
Acute phase response signaling	6.62E+00	4.48E-01	2.56
Estrogen receptor signaling	6.53E+00	4.77E-01	NaN
Adipogenesis pathway	6.31E+00	4.67E-01	NaN
FXR/RXR activation	6.03E+00	4.60E-01	NaN
Regulation of eIF4 and p70S6K signaling	5.86E+00	4.41E-01	0.35
mTOR signaling	5.62E+00	4.15E-01	−1.58
3-phosphoinositide biosynthesis	5.52E+00	4.11E-01	−1.07
Integrin signaling	5.51E+00	4.09E-01	−2.71
LXR/RXR activation	5.36E+00	4.53E-01	−3.68
Superpathway of inositol phosphate compounds	5.27E+00	3.94E-01	−1.98
D-myo-inositol-5-phosphate metabolism	5.06E+00	4.23E-01	−0.59
3-phosphoinositide degradation	4.86E+00	4.21E-01	−0.60
EIF2 signaling	4.60E+00	3.93E-01	2.61
RAN signaling	4.59E+00	7.37E-01	NaN
D-myo-inositol (1,4,5,6)-tetrakisphosphate biosynthesis	4.58E+00	4.23E-01	−0.13
D-myo-inositol (3,4,5,6)-tetrakisphosphate biosynthesis	4.58E+00	4.23E-01	−0.123
Unfolded protein response	4.44E+00	5.27E-01	NaN
Glioma invasiveness signaling	4.41E+00	4.93E-01	−1.52
Complement system	4.31E+00	5.79E-01	−2.14
Production of nitric oxide and reactive oxygen species in macrophages	4.29E+00	3.95E-01	−2.29
Macropinocytosis signaling	4.13E+00	4.69E-01	−0.39
LPS/IL-1 mediated inhibition of RXR function	4.09E+00	3.83E-01	2.65
Phagosome formation	3.98E+00	4.20E-01	NaN

#### RXR Activation and Fat Metabolism

Modulewise analysis had indicated a major effect of *E. coli* challenge on lipid metabolism demonstrated by the most significantly enriched pathways for DEGs in the dominating module turquoise. When including all DEGs in the *E. coli* challenge in the enrichment analysis, the LXR/RXR Activation pathway was significantly enriched and had one of the lowest negative *z*-scores (*z*-score = −3.68), indicating a substantial downregulation of this pathway in *E. coli* challenged vs. control animals. This is in agreement with the positive activation score for the pathway LPS/IL-1 mediated Inhibition of RXR Function (*z*-score = 2.65). As presented in Supplementary Image 6, a downregulation of the key receptor RXRα gene expression has far-reaching consequences for genes involved in lipid and cholesterol metabolism, bile acid homeostasis, and organic anion transport. Coordinated interplay of the nuclear receptors LXR and RXR is known for sensitive fine-tuning of the balance and maintenance of the body fat and cholesterol homeostasis (50). This fits the observation of an enrichment of the pathways Adipogenesis and Fatty Acid *beta* Oxidation in response to *E. coli* intramammary challenge also detected for the *E. coli* challenge associated module turquoise. Furthermore, due to the effects on the pathway LPS/IL-1 mediated Inhibition of RXR Function, a link is also provided for an immune response to the intramammary pathogen challenge also seen in the turquoise module.

#### Decreased Expression of Key Genes in Bovine Energy Metabolism

In addition to canonical pathways associated with fat metabolism, we also observed a significantly differential expression of particular genes involved in biochemical pathways associated with energy metabolism (Supplementary Table 13, Supplementary Images 7, 8). This conclusive, coordinated accumulation of DEGs had not been detected by IPA analysis, but it resulted in highly significantly enriched KEGG pathways Citrate Cycle (*p*_adj_ < 0.40E-16) and Glycolysis/Gluconeogenesis (*p* < 0.5E-19). All genes encoding key enzymes in glycolysis/gluconeogenesis showed a lower transcription in the cows with intramammary *E. coli* challenge compared to controls (e.g., *PCK1, PCK2, ALDOB, FBP1, GPI, HK1, G6PC*, see Supplementary Image 7). In addition, important genes involved in mitochondrial energy metabolism (e.g., *PCCA, PCCB, CPT1A, CPT1B, PDK1, PDK2, PDK4*) and particularly in the citrate cycle (e.g., *IDH1, ACO1, SDHA, SDHB, FH*, see Supplementary Image 8) were significantly differentially expressed.

#### Acute Phase Response Signaling and Complement System

**Figure 8:**
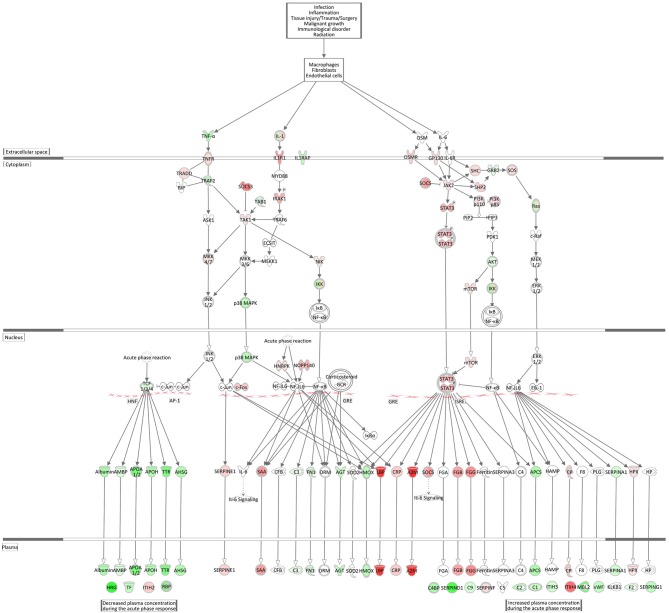
Gene expression of the acute phase response signaling in the *E. coli* challenged liver transcriptome compared to control hepatic transcriptome: green = lower expression and red = higher expression in *E. coli* challenged animals compared to untreated control (adapted from IPA, Qiagen).

Acute Phase Response Signaling, an essential component within the innate immune responses, was within the top significantly enriched Ingenuity canonical pathways and also had one of the highest activation scores in the hepatic transcriptome comparing *E. coli* challenged to non-challenged cows (*z*-score = 2.56). Figure 8 displays the very coordinated activation of this pathway in response to intramammary *E. coli* challenge with an increased expression of key cytokine receptor genes (*TNFR, IL1R1, OSMR, GP130*).

**Figure 9:**
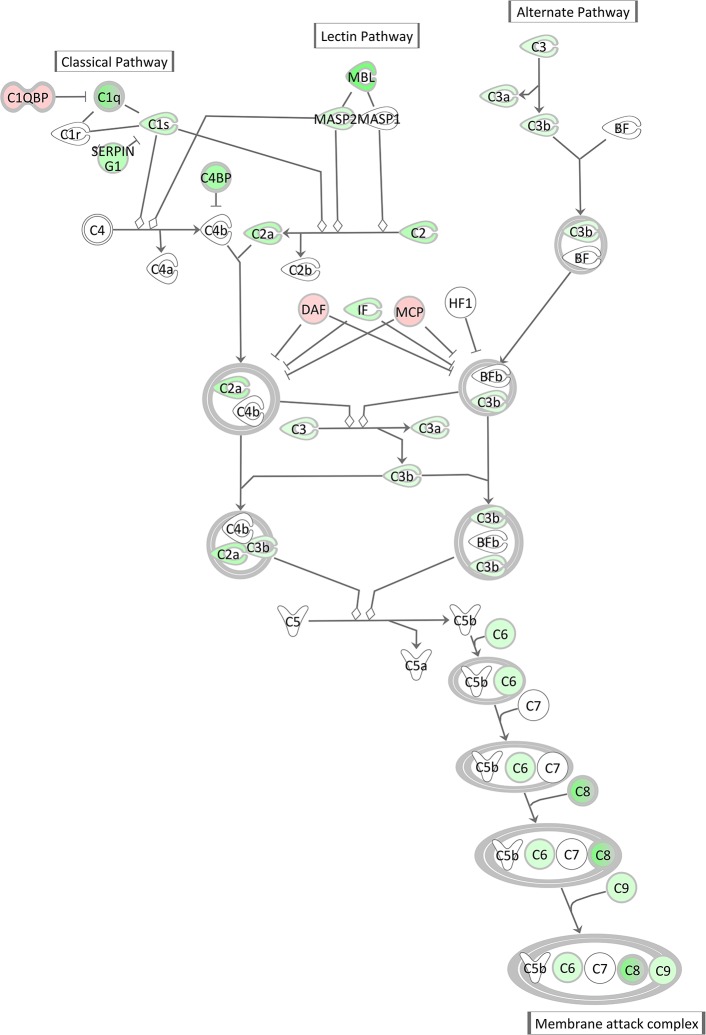
Gene expression of the complement system in the hepatic transcriptome of *E. coli* challenged animals compared to the control liver transcriptome: green = lower expression and red = higher expression in *E. coli* challenged animals compared to untreated control (adapted from IPA, Qiagen).

The Complement System pathway, which is as well an integral part of the innate immune system acting as bridge between innate and acquired immunity, was also highly significantly enriched with significantly DEGs after *E. coli* challenge. However, surprisingly and in contrast to the acute phase response pathway it displayed a low negative *z*-score indicating its strong and coordinated inactivation in *E. coli* challenged animals (*z*-score = −2.14). As shown in Figure 9, the vast majority of complement factors were significantly transcriptionally downregulated in the livers of *E. coli* challenged cows compared to the control, while in contrast some inhibitors of the complement system (*C1QBP, DAF/CD55, MCP/CD46*) were correspondingly upregulated in the challenged animals.

### Analysis of Potential Upstream Regulators in the Liver in Response to Intramammary *E. coli* Challenge

The IPA analysis predicted a large number of upstream regulators in response to intramammary *E. coli* challenge (see Supplementary Table 12). Most prominently, HNF4, known as a major transcription factor in hepatocytes, stood out with extremely high significance (*p* < 8.06E-83) and a high negative activation score. Indication on inactivation was obtained also for other known regulators of liver (lipid) metabolism (SREBF2, SREBF1, PPARG). This has already been demonstrated by the results from the module turquoise. This module comprised the largest number of genes and showed a strong correlation of module membership of genes and gene-infection association (see Figure 4). For the DEGs in this module, the enrichment analysis showed a strong association to amino acid and fatty acid metabolism and identified HNF4A as upstream regulator with a strongly predicted inactivation. The predicted inactivation of those factors was confirmed by a significantly differential expression of the respective genes themselves. Analogously, the positive activation score of *XBP1* and *ERN1*, which were on top of the list of genes with a predicted positive activation score, were also supported by a higher expression in the *E. coli* challenged animals compared to the controls. The XBP1 protein is a transcription factor that regulates the expression of genes important to the proper functioning of the immune system and in the cellular stress response (51). ERN1 (IRE1a) functions as a sensor of unfolded proteins in the endoplasmic reticulum and triggers an intracellular signaling pathway termed the unfolded protein response.

## Discussion

In summary, the results from the module-based expression analyses are very congruent to those obtained across the entire dataset. Our intramammary gland challenge experiment with two divergent mastitis pathogens revealed three key findings:

- Although *S. aureus* mastitis is commonly categorized as a local inflammation, we demonstrated that the liver is substantially affected via challenge of the udder displaying in part similar, in part opposite signatures of infection as mammary gland epithelial cells.
- *S. aureus* and *E. coli* dictate a pathogen-specific signature in the hepatic transcriptome after intramammary challenge.
- Both pathogens initiate activating and deactivating effects on the innate immune response of the liver after intramammary challenge.

### Intramammary *S. aureus*-Induced Challenge Triggers a Massive Hepatic Response

The liver is known to be central for both, metabolism and (innate) immune responses. Mammary gland challenge and infection by *S. aureus* is commonly considered a local, non-generalized process. Previous *S. aureus* challenge experiments with an analogous model had indicated no or only a moderate systemic host response (52) and no data were provided regarding liver involvement in this process. However, the fact that during the course of infection, the animals displayed an increase of body temperature with an average peak of >40.5°C (22) demonstrates that a clinical response to the intramammary *S. aureus* challenge had occurred. This is also documented by a massive change in liver function observed in the hepatic gene expression analysis. In our study, the biological pathway analysis of the hepatic transcriptome response to *S. aureus* intramammary challenge revealed that numerous significantly enriched pathways were affected such as the actin cytoskeleton and cell signaling (e.g., Epithelial Adherens Junction Signaling, ILK Signaling, Integrin Signaling, RhoGDI Signaling, ERK/MAPK Signaling, Actin Cytoskeleton Signaling, Signaling by Rho family GTPases, Remodeling of Epithelial Adherens Junctions, see Table 3, Supplementary Table 7). This indicates that multiple hepatic functions are affected by intramammary *S. aureus* challenge including lipid metabolism, innate immune response, citrate cycle, and gluconeogenesis. Although the pathogen challenge took place in the mammary gland, these crucial response effects to the invading pathogen were noted in the peripheral liver tissue.

Our study provides evidence that intramammary *S. aureus* challenge negatively affected hepatic pathways directly involved in combating the invading pathogen. This is already visible from the volcano plot of DEGs (Figure 3). The plot demonstrated a dominance of those particularly strongly DEGs (log2 Fold Change) that are lower expressed in pathogen challenged than in control animals compared to genes that are upregulated. For example, the Fcγ Receptor-Mediated Phagocytosis in Macrophages and Monocytes pathway (CD32) as well as the important cytokine/chemokine pathway IL8 Signaling (Table 3) were downregulated due to *S. aureus* challenge. The activation of Fcγ receptors favors the antibody-mediated phagocytosis of pathogens by macrophages or neutrophilic granulocytes, which is an essential part of the immune defense (53, 54). The downregulation of these hepatic processes suggest that such antibacterial mechanisms are attenuated in response to intramammary *S. aureus* challenge.

Furthermore, the pathway analysis revealed that Integrin Signaling had one of the lowest negative activation scores of all significantly enriched pathways, implying that this pathway was very substantially downregulated in hepatic tissue due to intramammary *S. aureus* challenge. Interestingly, β integrin genes showed a lower expression in the liver of *S. aureus* challenged cows compared to controls, in contrast to *alpha* integrin genes. In line with Integrin Signaling being downregulated in response to *S. aureus* challenge we also observed a downregulation of closely associated biological pathways, such as ILK Signaling and Actin Cytoskeleton signaling. Since ILK is at the center of cell-matrix adhesion and interacts with the cytoplasmic tail of *beta* integrins and couples it with the actin cytoskeleton, ILK signaling appears to be a central link between the affected pathways for innate immune responses against pathogenic invasion.

A further partner integrated into the biological and molecular crosstalk in response to intramammary *S. aureus* challenge is the pathway of Epithelial Adherens Junctions, which are crucial regulators of the actin cytoskeleton to maintain tissue homeostasis in critical cell processes and challenges that include tissue barrier function.

The central *S. aureus*-induced hepatic host response appears to be elicited by Actin Cytoskeleton signaling. The actin cytoskeleton is a dynamic structure necessary for cell and tissue organization, also comprising the epithelial barriers; thus, dynamic remodeling of the actin cytoskeleton is included as an essential component of a variety of cellular processes. For successful infection and replication, many pathogens are known to hijack the cytoskeleton using effector proteins introduced into the host cytosol by specialized secretion systems (55). This is associated with processes involving actin and actin-binding proteins in spatiotemporal actin polymerization/depolymerization processes. The rearrangement of the cytoskeleton and adhesion complexes linked to cell migration appears to be a major host structural component that is obviously manipulated by extracellular pathogens to defend against the infection. Interestingly, enhanced rather than downregulated actin signaling and actin-cytoskeleton rearrangement was recently described to occur after short term (3 h) mammary infection with *S. aureus* and was suggested to be involved in internalization of *S. aureus* 1027 into mammary epithelial cells (56).

### *S. aureus* and *E. coli* Elicit a Pathogen-Specific Signature in the Hepatic Transcriptome After Intramammary Challenge

The pathogen-specific signatures were initially demonstrated by modules of genes associated predominantly to a specific pathogen challenge. Subsequent pathway analyses revealed that particular biological processes are affected depending on the pathogen type, *S. aureus* or *E. coli*. Intramammary *S. aureus* challenge displayed that specific pathways predominantly connected to the cellular actin cytoskeleton were compromised in the liver transcriptome, e.g., ILK Signaling or Actin Cytoskeleton Signaling. The RhoGDI signaling, signaling by Rho family GTPases and ERK/MAPK signaling also fit into this cellular and molecular crosstalk in response to intramammary *S. aureus* challenge. Both, Rho family GTPases (including Rac, CDC42, and Rho) and classical extracellular activated kinase ERK1/2 MAP kinases have been identified as key integrators triggering cytoskeleton rearrangements. Signals from chemokine receptors and integrin molecules that coordinate these processes are transmitted to Rho family GTPases and passed on to cytoskeletal target proteins, which leads to the initiation of numerous processes including cell migration, morphogenesis, cytokinesis, and endo/exocytosis (57). Examples of DEGs in our study from these pathways with effects on the actin cytoskeleton are *MYL9, PIEZO1, FLNA, RALBP1, RIN3, MYOC1C*, and *CDH5* (58–64). All these genes were significantly lower expressed in the livers of *S. aureus* challenged animals. Simultaneously, genes included in RhoGDI signaling, the inhibitor of Rho family GTPases and binding partner that can tightly control Rho GTPases, are substantially higher expressed in *S. aureus* challenged compared to control cows. This again supports our hypothesis that there is a central effect of intramammary *S. aureus* infection on the hepatocyte actin cytoskeleton.

Remarkably, this pathway was significantly enriched only in the liver transcriptome in response to intramammary *S. aureus* challenge, but not after the *E. coli* challenge, which is in line with previous data in the mammary gland (56). This provides evidence that this pathway is specifically modulated during *S. aureus* challenge. It has been known that several *S. aureus* virulence factors can affect Rho GTPase activity in humans (65–69). Some of these factors such as SCIN, CHIP, and EDIN A-C are human specific and not encoded by the bovine isolate *S. aureus* 1027 used in this study (10). For others like the superantigen like proteins Ssl5, Ssl7, and Ssl10, the respective coding sequences have been detected in the genome of *S. aureus* 1027 (10). Both Ssl5 and Ssl10 interfere with Rho GTPase activity by binding G protein-coupled receptors (GPCRs) (70, 71). Besides, Ssl5 increased binding of chemokines to cells independent of chemokine receptors through their common glycosaminoglycan-binding site (70). For Ssl7 it has been shown that it targets C5 and thereby inhibits complement-mediated cell lysis possibly by blocking C5b-9 formation (72). The precise role of these proteins during interaction of *S. aureus* with the bovine host and in development of bovine mastitis, however, has still to be clarified.

While for *S. aureus*, the particular pathogen component responsible for the immune response is yet unclear, this is different for *E. coli*. For *E. coli*, LPS is known to be the key pathogen associated molecular pattern that is predominantly responsible for the inflammatory response to pathogen challenge (73). This is also confirmed by LPS being one of the most significantly predicted upstream regulators of DEGs in the module turquoise associated with *E. coli* challenge. Initiated by LPS, an entire cascade of LXR/RXR mediated signaling seems to be elicited (Supplementary Image 6).

Intramammary *E. coli* challenge also triggered a significant hepatic transcriptome response, but this was different to challenge by *S. aureus*. The function of LXR/RXR was significantly downregulated in challenged animals. This adaptation of the hepatic transcriptome seems to be pathogen-specific for the *E. coli* challenge. Retinoid X receptors (RXRs) are class II nuclear receptors; this subgroup includes *inter alia* liver X receptors (LXRs) (74). While LXRβ is expressed ubiquitously, LXRα is liver associated (75). LXRs are also activated by altered forms of cholesterol (76) and are fundamental for the transcriptional control of lipid metabolism in the liver (74, 76). The module darkred harboring DEGs enriched for the Cholesterol Biosynthesis pathway provided data supporting effects on cholesterol synthesis in the liver by intramammary *E. coli* challenge.

In recent years, several reports (74–76) shed light on the interactions between RXRs and the immune system, and especially on the acute phase response to infection (APR) (74). The APR is the first line of defense of the innate immune system induced by e.g., infections, injuries, or neoplasia (77) and is one of the most prominent pathways being upregulated in the liver after intramammary lipopolysaccharide (LPS) challenge (11, 18). Thus, the activated APR signaling observed in our hepatic transcriptome analysis in response to *E. coli* challenge is in accordance with data from the literature. Moreover, *FGG, FGB*, and *A2M* genes involved in APR signaling, were significantly upregulated in our analysis of DEGs.

In addition to its immunological effects, the APR has negative metabolic consequences: In lipid metabolism the APR leads to increased lipolysis, decreased fatty acid oxidation, and inhibition of bile acid synthesis (78). The RXRs seem to be a link between immunological and metabolic adaptations after infection. *E. coli* LPS as well as proinflammatory cytokines TNF*alpha* and IL1*beta* decreased the abundance of RXR proteins in the liver of hamsters (74). In a bovine cell model, Wang et al. (75) found that a synthetic LXR agonist decreased the synthesis of inflammatory cytokines in bovine mammary epithelial cells (bMECs) after challenge with *E. coli* LPS. Moreover, using transcriptome analysis of liver and mammary gland tissue samples following intramammary *E. coli* challenge, Moyes et al. (11) found that an increase in the expression of APR genes was accompanied by a decrease of key metabolic enzymes, including those of the lipid metabolism. Analogous data were obtained in our study indicating impaired gluconeogenesis and mitochondrial energy metabolism (Supplementary Table 13, Supplementary Images 7, 8), which are important metabolic processes in the lactating dairy cow. Respective differences in gene expression for *S. aureus* challenged cows were not observed: neither e.g., *PCK1, HK1, IDH1*, nor *CPTA* displayed significant differences between challenged and non-challenged cows.

These studies in the literature and our results from the hepatic transcriptome analysis consistently show that particularly *E. coli* mastitis is not only to be considered at the immunological level, but also in the context of metabolic changes. This is especially essential in case of severe clinical *E. coli* mastitis. Particularly the early lactation is a sensitive time period for mastitis, a time in which the liver is eventually confronted with the double burden of (bacterial) infection and increased metabolic demands for milk production. The aspect of an increased metabolic challenge of the animal due to pathogen-specific adaptions of the hepatic transcriptome might be a new approach for the treatment and prevention of the disease via direct interventions not only directly at the mammary gland, but also at systemic level.

### Both Pathogens Initiate Activating and Deactivating Effects on the Innate Immune Response in the Liver After Intramammary Challenge

There was a remarkable effect of *E. coli* challenge on the hepatic expression of the complement system genes. This comprised a lower expression of direct components of the complement system and a higher expression of genes encoding inhibitors of the complement system (*C1QBP, DAF/CD55, MCP/CD46*) in the challenged animals. Similar to the APR, the complement system is a powerful weapon of the innate immune response to fight against invading pathogens (79). However, our transcriptional analysis of livers after intramammary *E. coli* challenge revealed that the complement system [particularly its C1q (e.g., antigen-specific) governed branch] is incapable to react accordingly to the pathogen challenge while the APR seems to be activated, although with some balancing processes.

Günther et al. (80) challenged four first lactation Holstein cows with *E. coli* 1303 (the same strain as used in our experiment) and analyzed mRNA from udder tissue on a microarray platform. They also found a significant transcriptional downregulation of some components of the complement system, especially the first component of the classical pathway (C1 complex) and components in the membrane attack complex (MAC). Interestingly, Günther et al. also found complement inhibitors, such as *DAF* (decay-accelerating factor 1) and *C1QBP* (complement C1q-binding protein), to be increased in infected mammary gland tissue. Our data indicate that this effect is also reflected in the liver, the main tissue synthesizing complement system components. Based on these data, we suggest that while intramammary *E. coli* challenge triggers transcriptional processes of the APR in the liver, it also induces in parallel an active shut-down of the complement system already at transcript level.

The *E. coli* 1303 strain used in our study was studied by Leimbach et al. (81, 82), who reported that this strain does not carry many known virulence factors, which are thought to be advantageous to a mastitis pathogen. However, Abreu and Barbosa (79) reviewed various mechanisms and strategies of human pathogenic *E. coli* strains that might be used to either passively or even actively inhibit and overcome the damaging attacks of the complement system. The shutdown of the complement system as observed in our study drastically weakens the innate immune response and partially disarms the infected organism. This might be an important aspect of the pathogenesis of *E. coli* induced mastitis and might be responsible for the severe course of the disease. Alternatively, the shutdown of the complement system could represent an, albeit dangerous, regulatory mechanism to counterbalance the massive activation of the APR and its subsequent consequences.

The balanced activation of the immune system in the liver is also well reflected by alterations at transcriptional level of genes involved in different parts of the APR. While *TNFalpha* and *IL1beta* showed a lower expression in the hepatic transcriptome of intramammary *E. coli* challenged cows compared to control cows, the respective receptor genes (*TNFR* and *IL1R1*) displayed an inverse ratio of expression levels. In addition, while obviously major acute phase protein genes (*AM2, LBP*) were upregulated via *STAT3/STAT3A* in the course of challenge, *TCF1/3/4* was significantly lower expressed with correspondingly lower expression of genes encoding albumin (*ALB*), transferrin (*TF*), or histidine rich protein (*HRG*), a peptide with antimicrobial activity against *E. coli* (83).

In our pathway enrichment analysis, TGFB1 has been prioritized as potential regulator serving for the transmission of initial signals of e.g., β integrin and FcγR to the hepatic transcriptome response to intramammary *S. aureus* challenge. This suggests that TGFB1 could be a potential signaling driver of alterations in the hepatic transcriptome of pathogen challenged cows. Cross talk between TGFB1 and integrins is well described (84). *TGFB1* is the most important factor among the numerous cytokines and growth factors with various effects on immune cells (85). It prevents the immune system from attacking the body's own cells (85) by suppression of the immune system. The *TGFB1* gene expression has been reported to be involved in regulating immune response associated with experimentally induced *S. aureus* mastitis (13). In our study, its strong candidacy as key regulator of the immunomodulatory response to intramammary *S. aureus* challenge in the liver transcriptome is supported by its highly significant lower hepatic gene expression level in *S. aureus* infected cows compared to controls and by its highly significant predicted role as an inactivated upstream regulator.

In the hepatic transcriptome of intramammary *E. coli* infected cows, *TGFB1* expression levels were also significantly downregulated compared to the untreated controls. The inactivation of this immunosuppressor is also in line with the canonical pathway analysis of *E. coli* infected vs. controls, where the TGF beta Signaling pathway had a high positive *z*-score (3.02), indicating a considerable activation of this pathway in the livers of *E. coli* infected animals. Thus, the lower hepatic immunoinhibitor gene expression in response to *S. aureus* or *E. coli* intramammary challenge has initiated an activation of the immune response via upregulation of expression levels of target genes acting downstream in this pathway (e.g., *SMAD1, SMA5*). This supports a well and tightly orchestrated regulation of the immune response beyond the local site of bacterial infection.

Our experiment has proved that the liver is affected from an intramammary pathogen challenge with both *S. aureus* and *E. coli*, respectively. We found that both pathogens can attack the immune system in an important metabolic organ that is located at a considerable distance from the site of the animal's direct pathogen contact. Thus, only antibiotic therapy of cows suffering from mastitis might not be sufficient. Immunostimulant drugs could favor the course of the disease but would have to be used in a pathogen-specific manner. Especially for *E. coli* mastitis, the influences of the infection on the liver metabolism should not be neglected. Particularly in the early lactation, the cow's energy demand for milk production can hardly be covered by the energy consumed with the feed (12). An infection, which also leads to adaptations of important metabolic pathways in the liver (lipid metabolism, glycolysis/gluconeogenesis, mitochondrial energy metabolism), further increases this energy deficit. Therefore, energy supplementation (e.g., glucose) could be beneficial for cows suffering from *E. coli* mastitis.

### Potential Impact of Early Lactation Environmental Effects

Differences in the environment between the infection and control groups could have introduced a potential bias possibly impacting the interpretation of our study. Thus, we compared initially the metabolic status of the challenge groups and the control group prior to the intramammary pathogen challenge, which might have resulted in environmentally, but not challenge-induced hepatic differences between the groups. However, neither blood serum NEFA nor BHB provided indication for a significantly different metabolic status of the different groups. Furthermore, an overlay of the results from the differential expression analysis added further proof that the vast majority of expression differences were due to pathogen challenge. To this end, we also compared the Top 25 significantly enriched canonical pathways for two separate input gene lists in *E. coli* and *S. aureus* challenge (Supplementary Tables 14, 15, Supplementary Image 9): List 1 comprises genes differentially expressed when comparing the *E. coli* group vs. control but not differentially expressed in *S. aureus* vs. control and list 2 *vice versa*. If environment indeed had been the main driver of the observed hepatic transcriptome effects, few and similar pathways should have resulted from the two lists. However, the comparison of the pathways enriched in the analysis of *E. coli* or *S. aureus* challenged animals, respectively, vs. control showed distinct differences in affected canonical pathways or their activation status (Supplementary Image 9). In addition to our cluster-based module analysis, this provides further evidence that indeed pathogen response and not e.g., divergent environmentally induced metabolic status had driven the transcriptomic differences between pathogen challenge group and control.

## Conclusions

Both, *S. aureus* and *E. coli* elicited systemic effects on the host after intramammary challenge and seem to use pathogen-specific targeting strategies to bypass the innate immune system: While *S. aureus* inhibits the cell signaling via integrin, FcγR and Rho GTPases in the liver, *E. coli* switches the complement system off. Also metabolic hepatic pathways (e.g., lipid metabolism or gluconeogenesis and citrate cycle) are affected after mammary gland challenge, demonstrating that the liver reduces metabolic tasks in favor of the predominant immune response after infection. Therefore, a revised, pathogen-specific treatment regime for mastitis, which goes beyond mere antibiotic therapy, might be beneficial for the course of the infection.

The most striking result of our study is that we demonstrate for the first time that *S. aureus* udder challenge causes an immune response beyond the original local site of the mastitis. We found that in the peripheral liver tissue defined biological pathways are switched on/off in a coordinated manner to balance the immune response in the entire organism. TGFB1 signaling plays a crucial role in this context.

## Data Availability Statement

RNA-Seq datasets are submitted to the ENA repository (https://www.ebi.ac.uk/ena, Project number PRJEB33849, accession numbers ERR3466640 - ERR3466680) at EMBL-EBI.

## Ethics Statement

The animal study was reviewed and approved by the FBN cohort, the experiment was conducted under the reference number 7221.3-1-055/15 with the approval by the responsible authority (LALLF, Landesamt für Landwirtschaft, Lebensmittelsicherheit und Fischerei Mecklenburg-Vorpommern, Rostock, Germany). For the TiHo cohort, the experiment was performed under the reference number 33.12-42502-04-15/2024 by the Lower Saxony Federal State Office for Consumer Protection and Food Safety. This study was submitted to and approved by the ethics committees of the Leibniz Institute for Farm Animal Biology and the University of Veterinary Medicine Hanover, respectively. All ethical evaluations were performed as required by the German Animal Care law and associated legislative regulations (23).

## Author Contributions

RW, H-MS, WP, HZ, MH, H-JS, MS, SE, and CK designed research. AH, JB, MM, and LR performed research. AH, RW, DB, and CK analyzed data and wrote the paper. All authors read and approved the final manuscript.

## Conflict of Interest

The authors declare that the research was conducted in the absence of any commercial or financial relationships that could be construed as a potential conflict of interest.
