# Supplementary figures and images for: Hepatic Transcriptome Analysis Identifies Divergent Pathogen-Specific Targeting-Strategies to Modulate the Innate Immune System in Response to Intramammary Infection
Source: Front Immunol. 2020 Apr 29;11:715. doi: 10.3389/fimmu.2020.00715 (PMC7202451; doi:10.3389/fimmu.2020.00715)

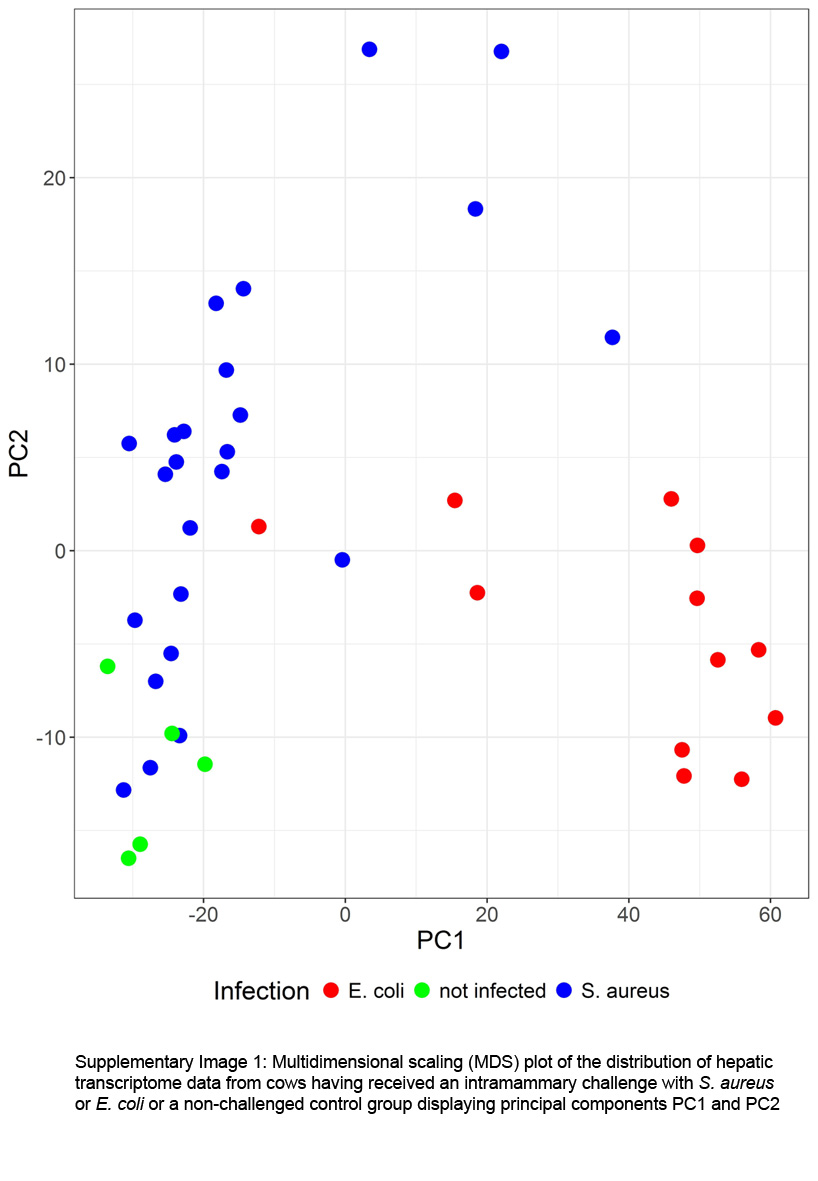

Supplement: Supplementary file 1 [file Image_1.jpg]

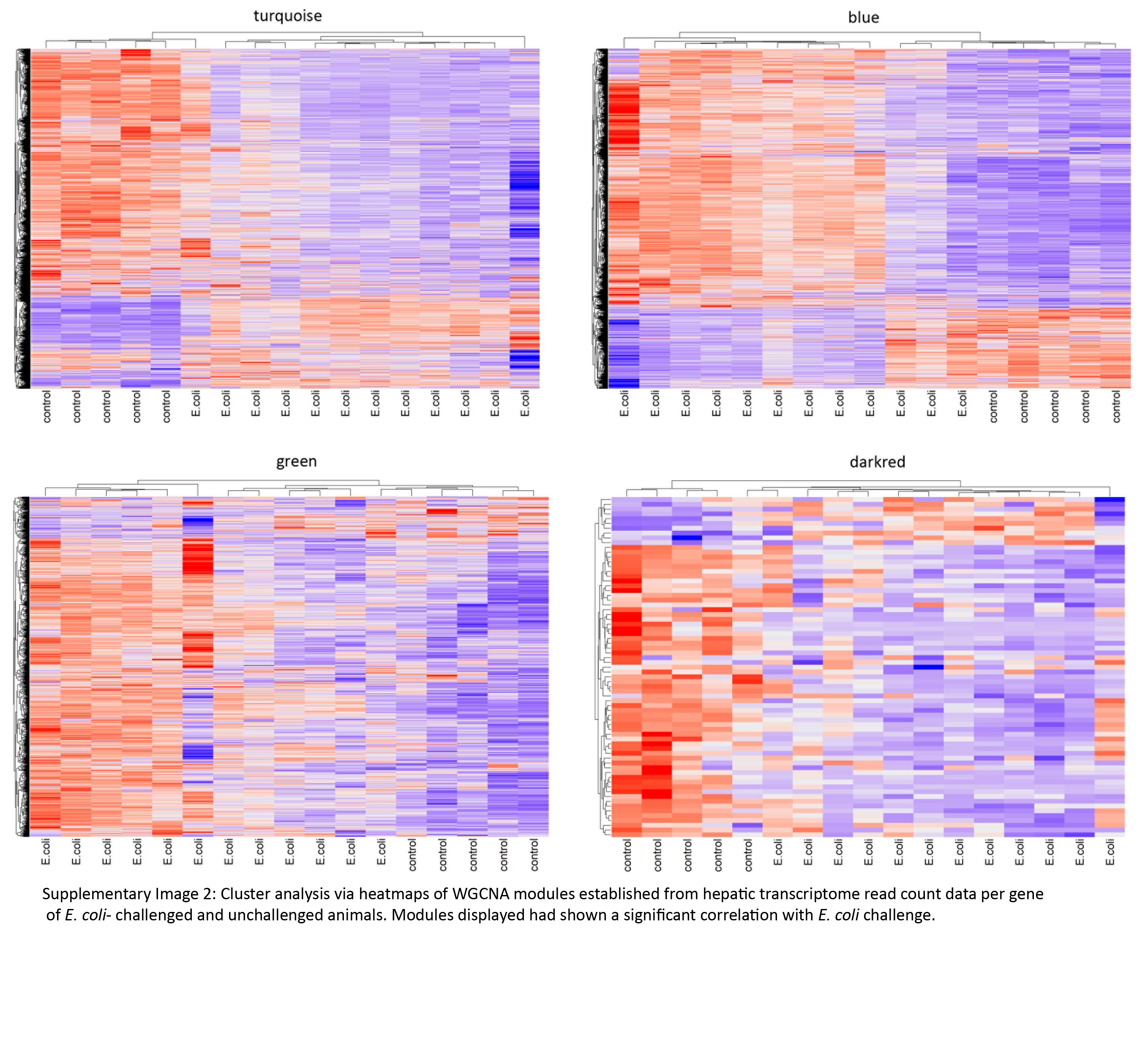

Supplement: Supplementary file 2 [file Image_2.jpg]

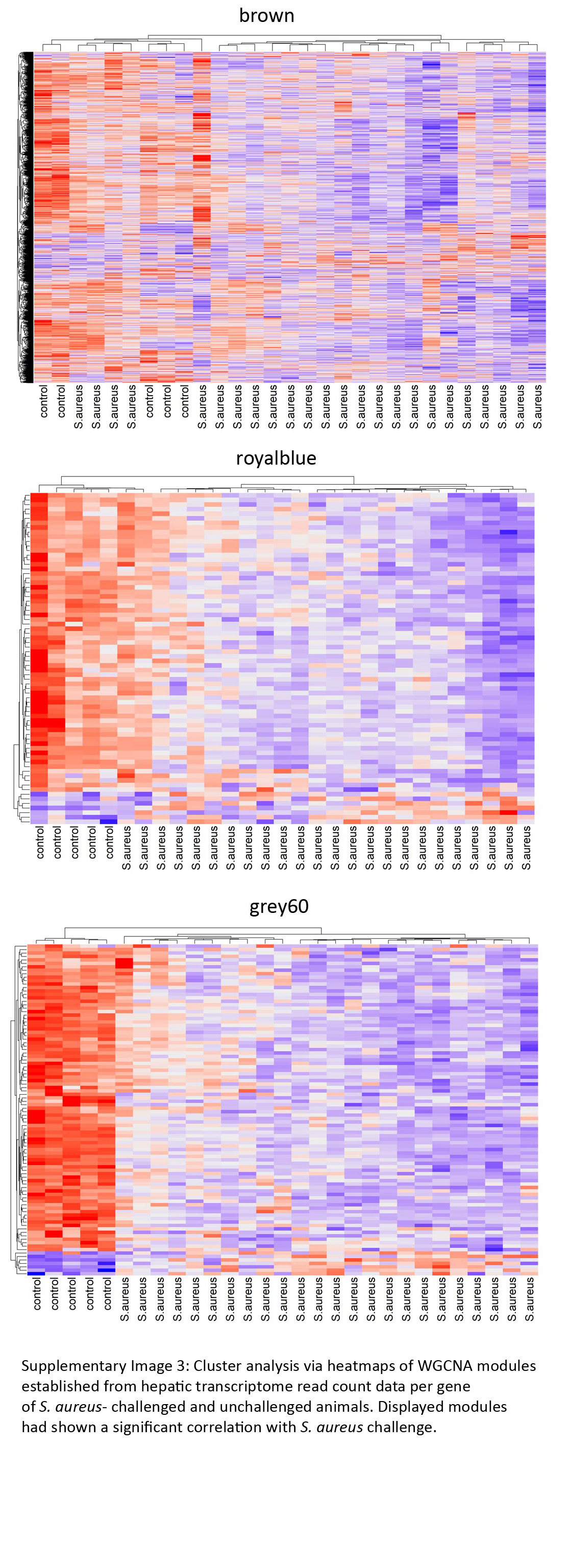

Supplement: Supplementary file 3 [file Image_3.jpg]

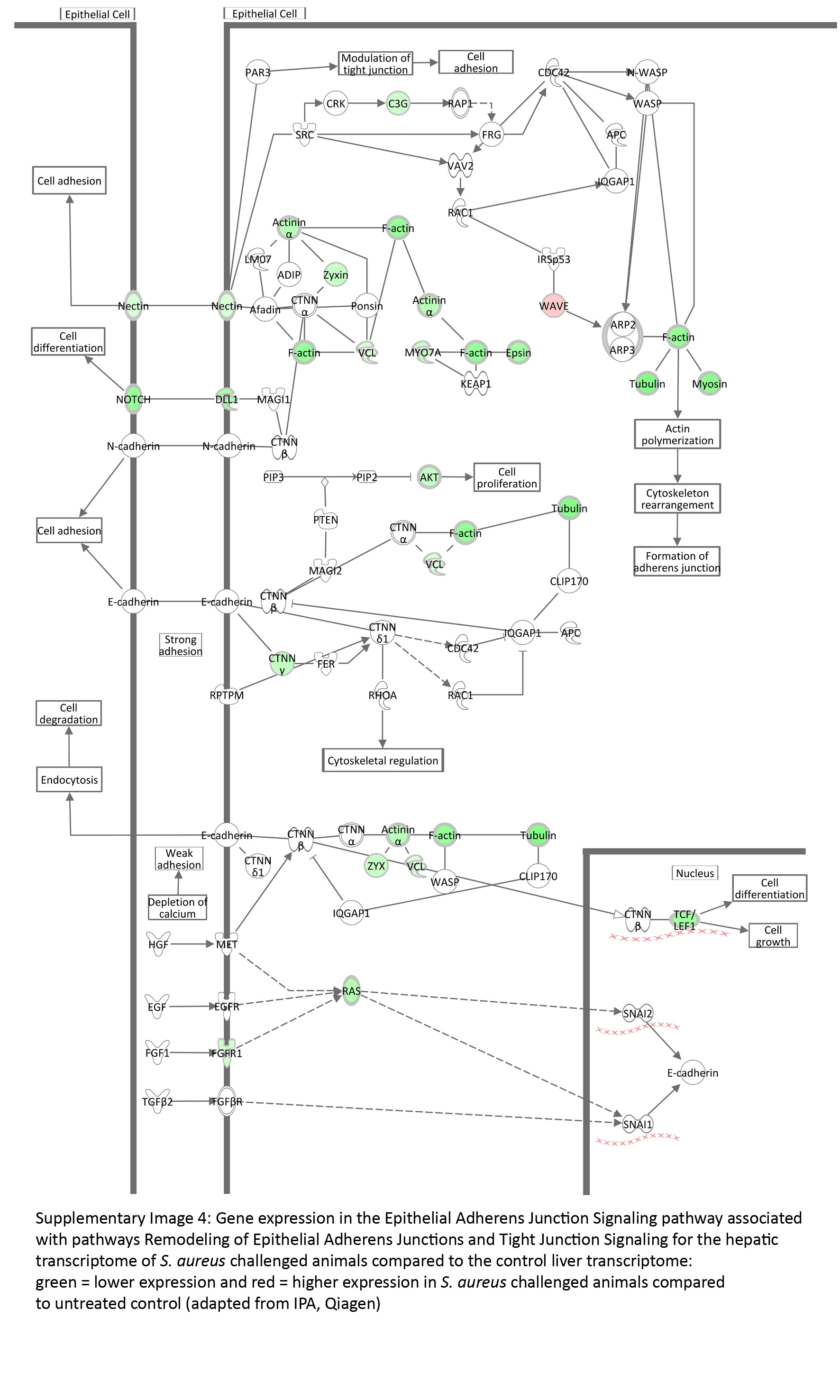

Supplement: Supplementary file 4 [file Image_4.jpg]

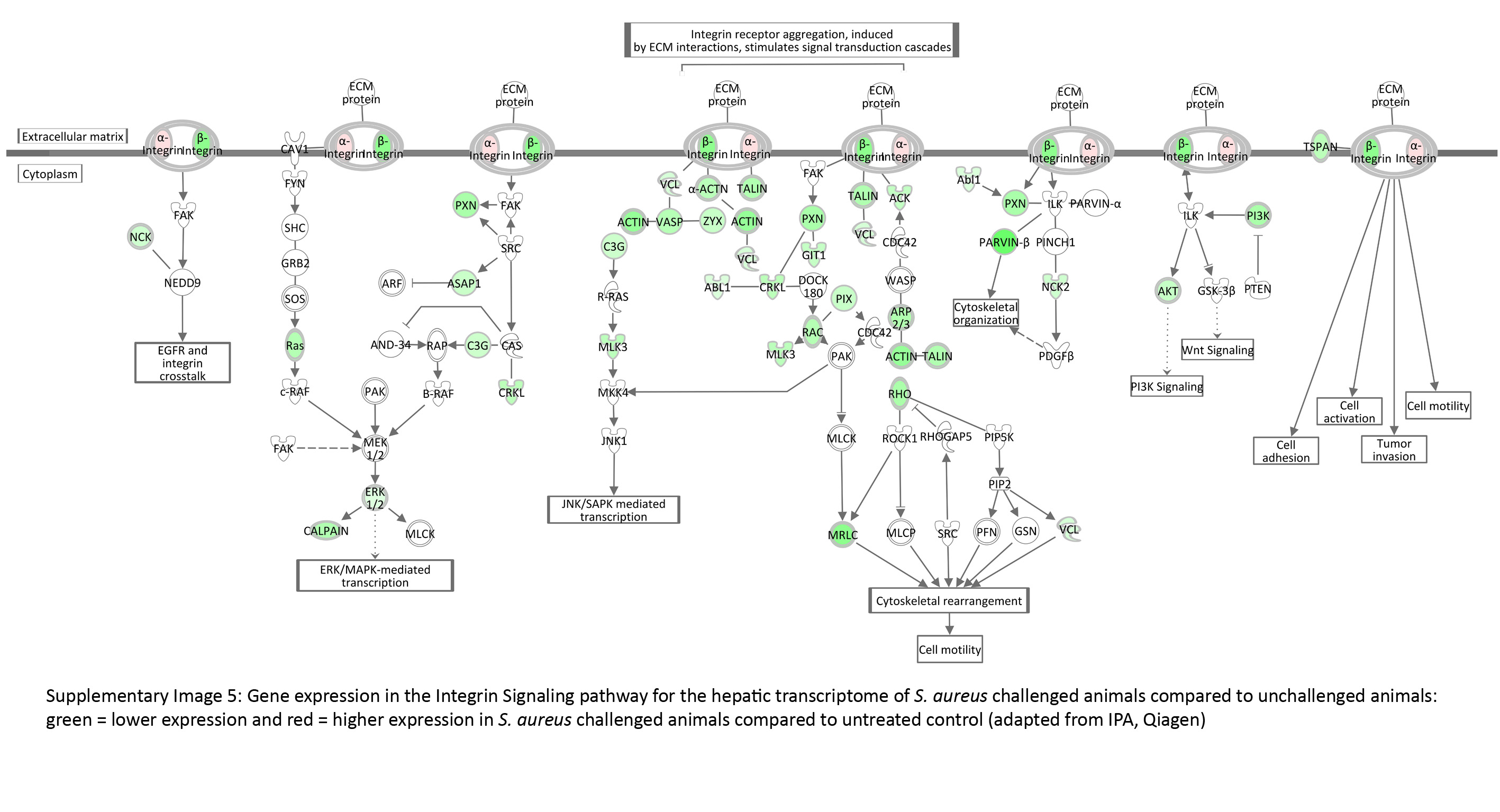

Supplement: Supplementary file 5 [file Image_5.jpg]

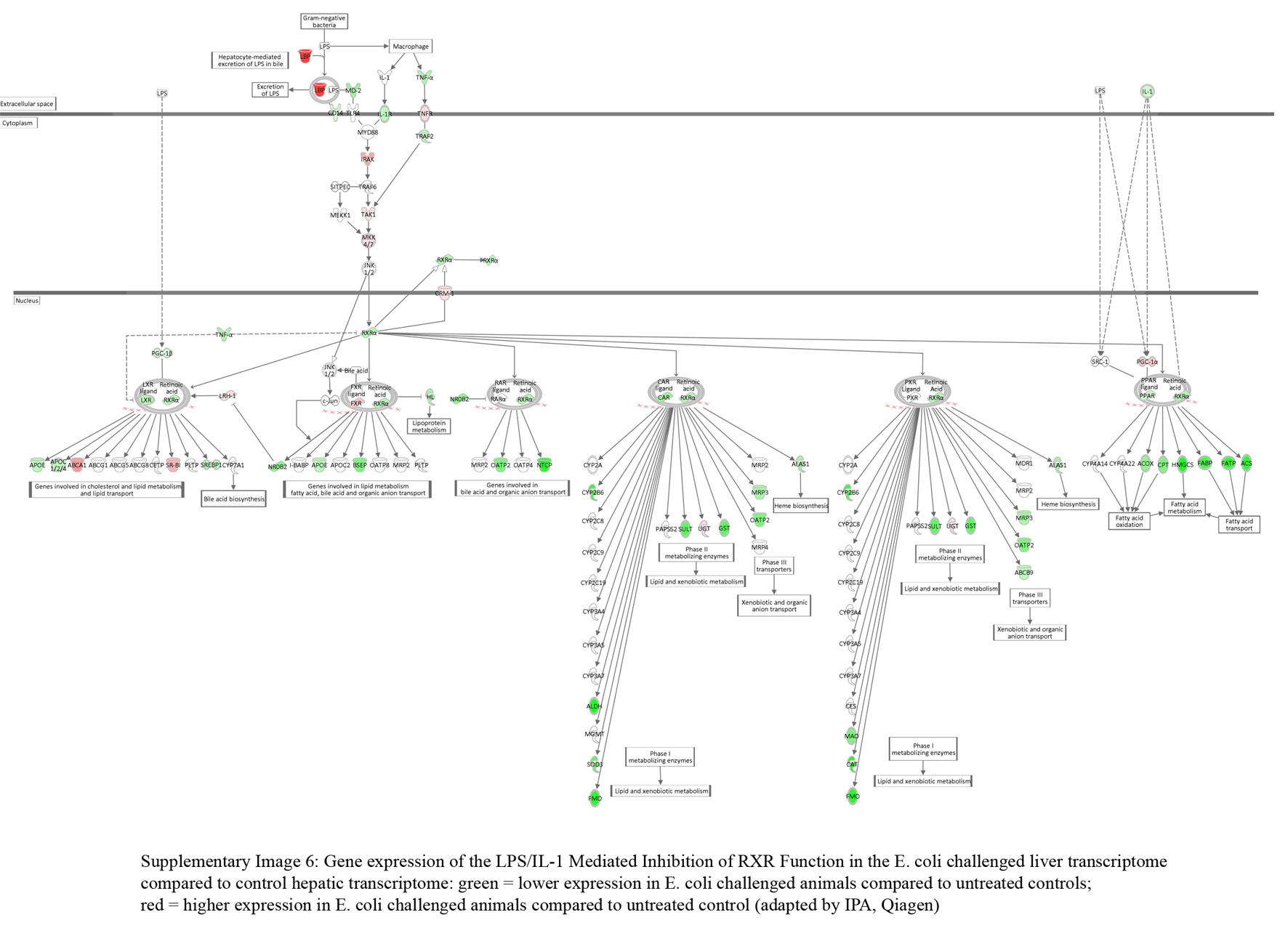

Supplement: Supplementary file 6 [file Image_6.jpg]

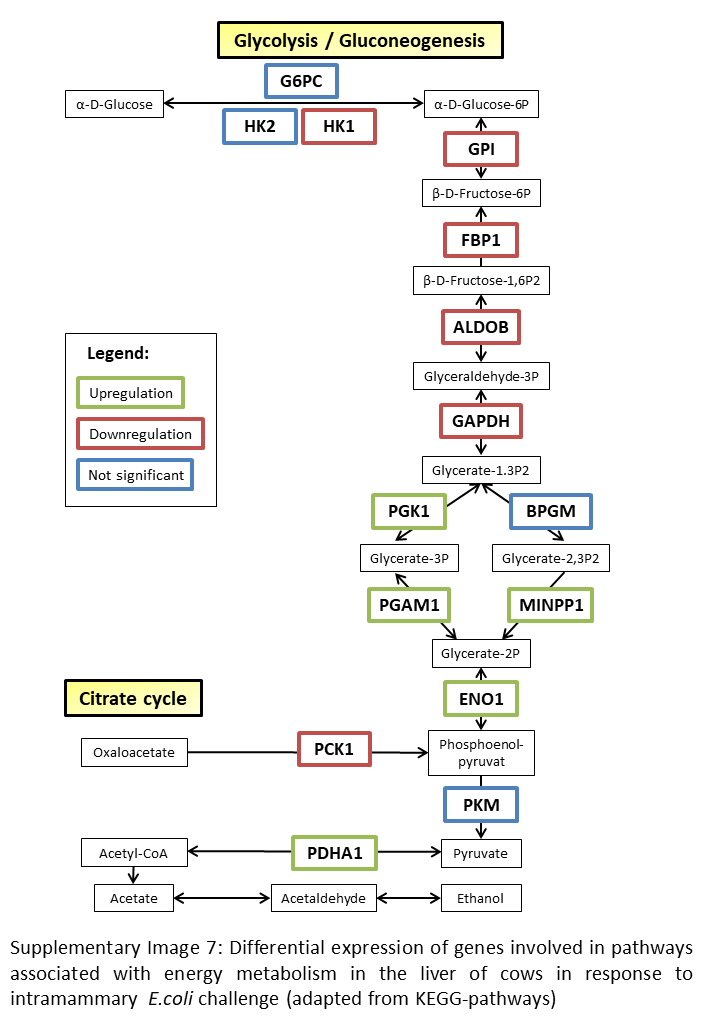

Supplement: Supplementary file 7 [file Image_7.tif]

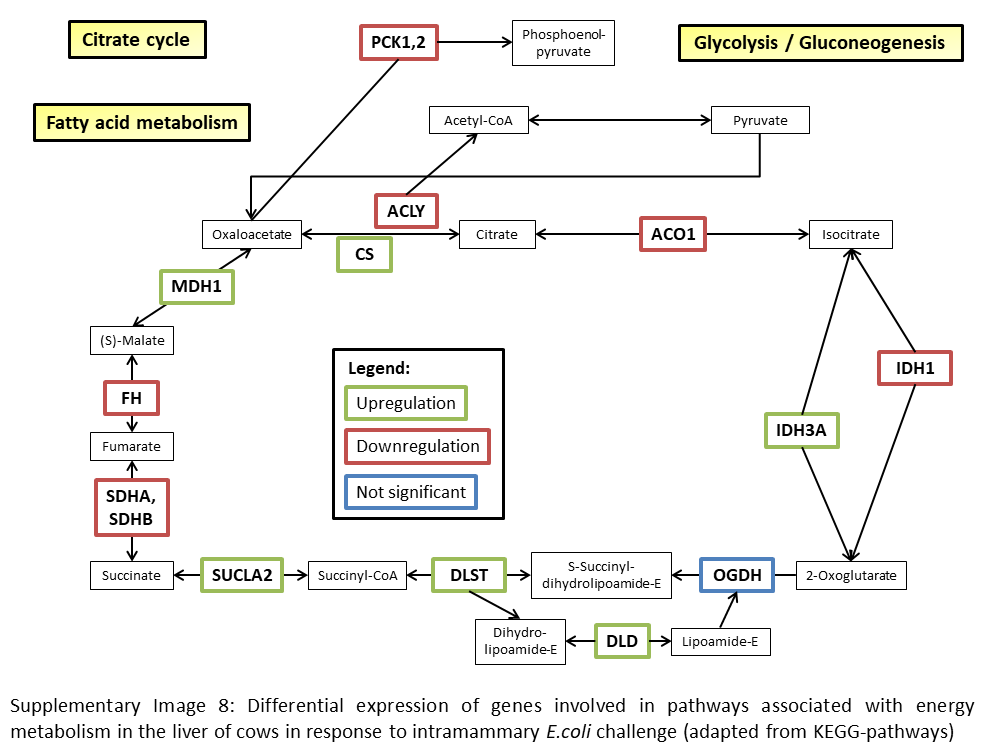

Supplement: Supplementary file 8 [file Image_8.tif]

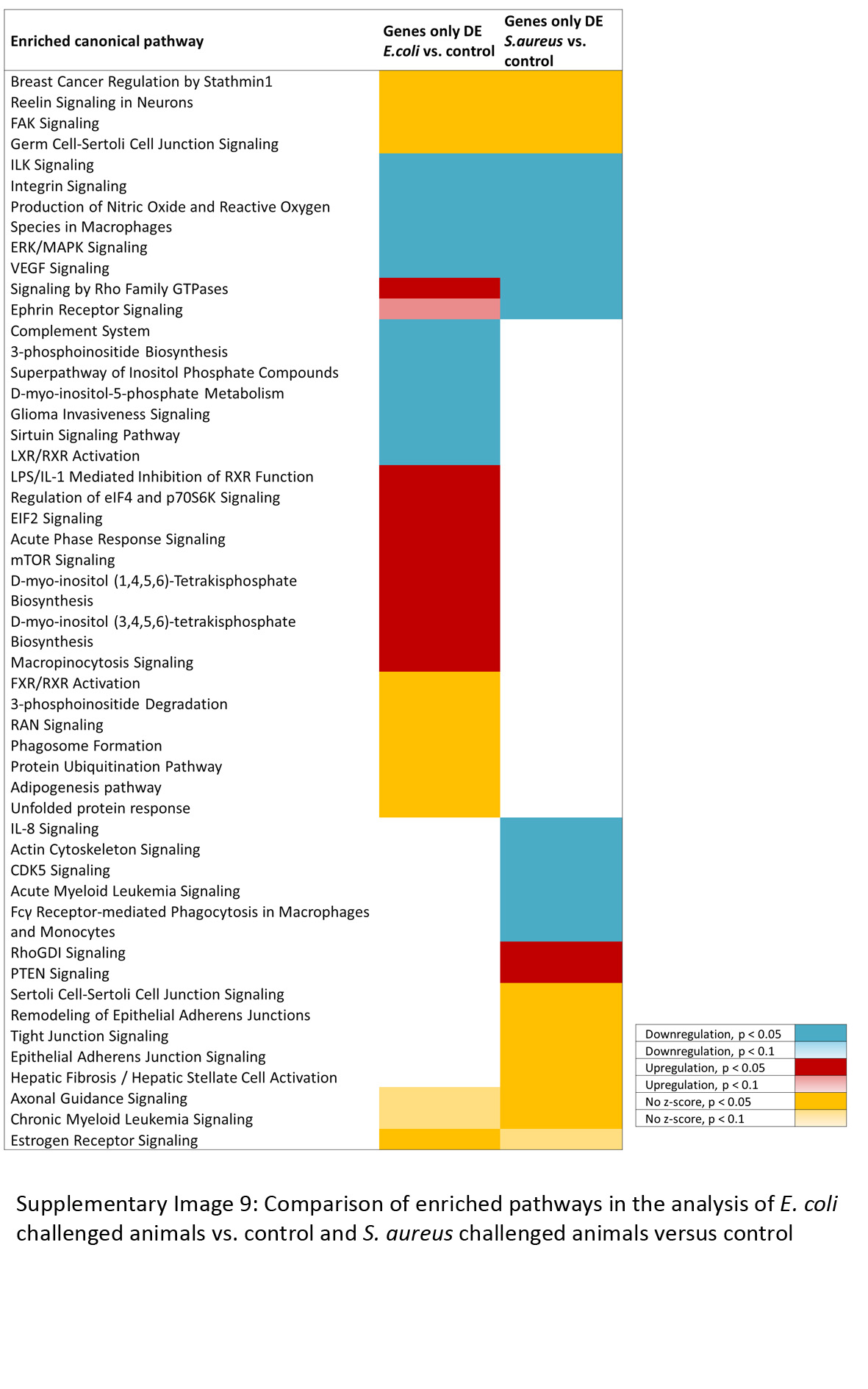

Supplement: Supplementary file 9 [file Image_9.jpg]
