# Supplementary material for: Hepatic Transcriptome Analysis Identifies Divergent Pathogen-Specific Targeting-Strategies to Modulate the Innate Immune System in Response to Intramammary Infection
Source: Front Immunol. 2020 Apr 29;11:715. doi: 10.3389/fimmu.2020.00715 (PMC7202451; doi:10.3389/fimmu.2020.00715)
Supplement: Supplementary file 23 [file Table_14.docx]

Supplementary Table 14: Top 25 significantly enriched Ingenuity canonical pathways for *S. aureus*-specific differentially expressed loci compared to the control.

| Ingenuity Canonical Pathways | -log  (p-value) | Ratio | z-score |
| --- | --- | --- | --- |
| Epithelial Adherens Junction Signaling | 5.27E+00 | 1.12E-01 | NaN |
| CDK5 Signaling | 5.14E+00 | 1.28E-01 | -1.069 |
| Remodeling of Epithelial Adherens Junctions | 5.07E+00 | 1.59E-01 | NaN |
| Acute Myeloid Leukemia Signaling | 4.78E+00 | 1.27E-01 | -2.111 |
| ERK/MAPK Signaling | 4.54E+00 | 9.13E-02 | -0.688 |
| Signaling by Rho Family GTPases | 4.31E+00 | 8.27E-02 | -3.441 |
| ILK Signaling | 4.28E+00 | 9.05E-02 | -1.698 |
| RhoGDI Signaling | 4.22E+00 | 9.29E-02 | 2.324 |
| FAK Signaling | 3.87E+00 | 1.11E-01 | NaN |
| Breast Cancer Regulation by Stathmin1 | 3.80E+00 | 8.29E-02 | NaN |
| Opioid Signaling Pathway | 3.78E+00 | 7.78E-02 | -1.342 |
| B Cell Receptor Signaling | 3.77E+00 | 8.54E-02 | -2.668 |
| Actin Cytoskeleton Signaling | 3.33E+00 | 7.59E-02 | -3.153 |
| Integrin Signaling | 3.26E+00 | 7.73E-02 | -2.84 |
| Protein Kinase A Signaling | 3.24E+00 | 6.30E-02 | 2.065 |
| Germ Cell-Sertoli Cell Junction Signaling | 3.23E+00 | 8.24E-02 | NaN |
| Ephrin Receptor Signaling | 3.23E+00 | 8.24E-02 | -3.051 |
| PTEN Signaling | 3.22E+00 | 9.45E-02 | 2.887 |
| Axonal Guidance Signaling | 3.18E+00 | 6.05E-02 | NaN |
| Tight Junction Signaling | 3.13E+00 | 8.38E-02 | NaN |
| CD40 Signaling | 3.12E+00 | 1.14E-01 | -2.121 |
| VEGF Signaling | 3.04E+00 | 9.57E-02 | -2.111 |
| Synaptic Long Term Potentiation | 3.01E+00 | 8.96E-02 | -0.577 |
| Sertoli Cell-Sertoli Cell Junction Signaling | 3.00E+00 | 7.81E-02 | NaN |
| AMPK Signaling | 2.99E+00 | 7.30E-02 | -1.604 |
