# Supplementary material for: Hepatic Transcriptome Analysis Identifies Divergent Pathogen-Specific Targeting-Strategies to Modulate the Innate Immune System in Response to Intramammary Infection
Source: Front Immunol. 2020 Apr 29;11:715. doi: 10.3389/fimmu.2020.00715 (PMC7202451; doi:10.3389/fimmu.2020.00715)
Supplement: Supplementary file 24 [file Table_15.docx]

Supplementary Table 15: Top 25 significantly enriched Ingenuity canonical pathways for *E. coli*-specific differentially expressed loci compared to the control.

| Ingenuity Canonical Pathways | -log  (p-value) | Ratio | z-score |
| --- | --- | --- | --- |
| Protein Ubiquitination Pathway | 8.24E+00 | 3.68E-01 | NaN |
| D-myo-inositol-5-phosphate Metabolism | 7.20E+00 | 3.96E-01 | -0.367 |
| 3-phosphoinositide Biosynthesis | 7.13E+00 | 3.74E-01 | -0.671 |
| Sirtuin Signaling Pathway | 7.10E+00 | 3.42E-01 | -2.194 |
| Superpathway of Inositol Phosphate Compounds | 6.88E+00 | 3.55E-01 | -1.46 |
| D-myo-inositol (1,4,5,6)-Tetrakisphosphate Biosynthesis | 6.29E+00 | 3.93E-01 | 0.13 |
| D-myo-inositol (3,4,5,6)-Tetrakisphosphate Biosynthesis | 6.29E+00 | 3.93E-01 | 0.130.13 |
| Acute Phase Response Signaling | 5.86E+00 | 3.71E-01 | 3.111 |
| 3-phosphoinositide Degradation | 5.77E+00 | 3.76E-01 | 0 |
| Regulation of eIF4 and p70S6K Signaling | 5.57E+00 | 3.71E-01 | 1.512 |
| FXR/RXR Activation | 5.47E+00 | 3.87E-01 | NaN |
| Estrogen Receptor Signaling | 5.42E+00 | 3.88E-01 | NaN |
| mTOR Signaling | 5.27E+00 | 3.47E-01 | 0.289 |
| Fatty Acid β-oxidation I | 4.94E+00 | 5.11E-01 | -3.545 |
| Role of Tissue Factor in Cancer | 4.20E+00 | 3.64E-01 | NaN |
| LPS/IL-1 Mediated Inhibition of RXR Function | 4.20E+00 | 3.26E-01 | 1.768 |
| Assembly of RNA Polymerase II Complex | 4.02E+00 | 4.60E-01 | NaN |
| EIF2 Signaling | 3.99E+00 | 3.22E-01 | 3.618 |
| RAN Signaling | 3.98E+00 | 6.32E-01 | NaN |
| Macropinocytosis Signaling | 3.84E+00 | 3.91E-01 | 0.408 |
| Glioma Invasiveness Signaling | 3.83E+00 | 4.03E-01 | -0.18 |
| LXR/RXR Activation | 3.59E+00 | 3.52E-01 | -4.003 |
| Unfolded protein response | 3.58E+00 | 4.29E-01 | NaN |
| ERK5 Signaling | 3.51E+00 | 3.97E-01 | 2.646 |
| Clathrin-mediated Endocytosis Signaling | 3.32E+00 | 3.14E-01 | NaN |
